# Parkinson mice show functional and molecular changes in the gut long before motoric disease onset

**DOI:** 10.1186/s13024-021-00439-2

**Published:** 2021-06-02

**Authors:** Manuela Gries, Anne Christmann, Steven Schulte, Maximilian Weyland, Stephanie Rommel, Monika Martin, Marko Baller, Ralph Röth, Stefanie Schmitteckert, Marcus Unger, Yang Liu, Frederik Sommer, Timo Mühlhaus, Michael Schroda, Jean-Pierre Timmermans, Isabel Pintelon, Gudrun A. Rappold, Markus Britschgi, Hilal Lashuel, Michael D. Menger, Matthias W. Laschke, Beate Niesler, Karl-Herbert Schäfer

**Affiliations:** 1 Department of Informatics and Microsystems and Technology, University of Applied Science Kaiserslautern, Working Group Enteric Nervous System, 66482 Zweibrücken, Germany; 2 Department of Human Molecular Genetics, University of Heidelberg, 69120 Heidelberg, Germany; 3 Department of Neurology, Saarland University, 66421 Homburg, Germany; 4 Molecular Biotechnology and Systems Biology, University of Kaiserslautern, 67663 Kaiserslautern, Germany; 5 Computational Systems Biology, University of Kaiserslautern, 67663 Kaiserslautern, Germany; 6 Laboratory of Cell Biology and Histology, Department of Veterinary Sciences, University of Antwerp, 2610 Antwerp, Belgium; 7 Interdisciplinary Center of Neuroscience, 69120 Heidelberg, Germany; 8 Roche Pharma Research and Early Development, Neuroscience and Rare Diseases Discovery and Translational Medicine Area, Neuroscience Discovery, Roche Innovation Center Basel, 4070 Basel, Switzerland; 9 Laboratory of Molecular and Chemical Biology of Neurodegeneration, Brain Mind Institute, École Polytechnique Fédérale de Lausanne, 1015 Lausanne, Switzerland; 10 Institute for Clinical & Experimental Surgery, Faculty of Medicine, Saarland University, 66421 Homburg, Germany; 11 Department of Pediatric Surgery, Medical Faculty Mannheim, University of Heidelberg, 68167 Mannheim, Germany

**Keywords:** Parkinson’s disease, Early onset, Enteric nervous system, Gastrointestinal motility, Protein-and miRNA biomarkers

## Abstract

**Background:**

There is increasing evidence that Parkinson’s disease (PD) might start in the gut, thus involving and compromising also the enteric nervous system (ENS). At the clinical onset of the disease the majority of dopaminergic neurons in the midbrain is already destroyed, so that the lack of early biomarkers for the disease represents a major challenge for developing timely treatment interventions. Here, we use a transgenic A30P-α-synuclein-overexpressing PD mouse model to identify appropriate candidate markers in the gut before hallmark symptoms begin to manifest.

**Methods:**

Based on a gait analysis and striatal dopamine levels, we defined 2-month-old A30P mice as pre-symptomatic (psA30P), since they are not showing any motoric impairments of the skeletal neuromuscular system and no reduced dopamine levels, but an intestinal α-synuclein pathology. Mice at this particular age were further used to analyze functional and molecular alterations in both, the gastrointestinal tract and the ENS, to identify early pathological changes. We examined the gastrointestinal motility, the molecular composition of the ENS, as well as the expression of regulating miRNAs. Moreover, we applied A30P-α-synuclein challenges in vitro to simulate PD in the ENS.

**Results:**

A retarded gut motility and early molecular dysregulations were found in the myenteric plexus of psA30P mice. We found that i.e. neurofilament light chain, vesicle-associated membrane protein 2 and calbindin 2, together with the miRNAs that regulate them, are significantly altered in the psA30P, thus representing potential biomarkers for early PD. Many of the dysregulated miRNAs found in the psA30P mice are reported to be changed in PD patients as well, either in blood, cerebrospinal fluid or brain tissue. Interestingly, the in vitro approaches delivered similar changes in the ENS cultures as seen in the transgenic animals, thus confirming the data from the mouse model.

**Conclusions:**

These findings provide an interesting and novel approach for the identification of appropriate biomarkers in men.

**Supplementary Information:**

The online version contains supplementary material available at 10.1186/s13024-021-00439-2.

## Introduction

In Parkinson’s disease (PD), the loss of nigrostriatal dopaminergic neurons causes the hallmark motor symptoms of muscle rigidity, tremor at rest, and bradykinesia [1]. In recent years, early non-motor symptoms of PD have gained attention; these include sleep disturbance, pain, depression, and constipation, and can occur more than 15 years before clinical motor symptoms manifest [2]. Histopathological examination of postmortem PD brains has revealed intraneuronal accumulations of misfolded α-synuclein, termed Lewy bodies [3]. These Lewy bodies disrupt protein ubiquitination, cytoskeleton reorganization, synaptic function and vesicle release, and increase oxidative stress levels to promote PD pathogenesis [4]. Lewy bodies are present not only in the central nervous system (CNS) but also in the enteric nervous system (ENS) of the intestine, so it is not only the brain but also the gut that is involved in PD pathogenesis [5, 6]. In 2003, Braak et al. postulated that neurotoxic α-synucleins may originate in the gut and migrate to the brain via the vagal nerve [7, 8]. This theory was recently supported by studies in animal models [9, 10], which confirmed that α-synuclein transport can be purged by vagal nerve resection [11, 12]. Previous research has shown that the crosstalk between the gut and the brain during PD pathogenesis is mainly influenced by intestinal dysbiosis [13, 14]. This suggests that the gastrointestinal (GI) motility deficits associated with PD, such as constipation, are caused by alterations in the microbial composition of the gut, which disrupt gut microbiome and gut-brain-axis may homeostasis and increase inflammation and permeability of the gut [14–17]. These findings indicate that PD originates in the gut, and that a dysregulated gut contributes to PD symptoms. However, changes in the ENS during PD onset have not been well-described. To better understand the role of the gut in PD, a more detailed investigation of the early phase of PD within the gut is essential. The current study aimed to assess early pathological changes associated with PD in the ENS to identify biomarkers of pre-clinical PD. In recent years, miRNAs have emerged as important regulators of gene expression, cell differentiation, cell maturation, apoptosis, and immune responses [18]. They are present in circulating body fluids [19] as well as gut mucosal tissues, so they can be investigated by colonoscopy [20]. Abnormal miRNA expression has been linked to many human diseases, and miRNAs have been identified as diagnostic and prognostic biomarkers for several disorders, including PD [21, 22]. However, no PD-specific miRNAs have been identified in the gut for the pre-clinical diagnosis of PD so far [23, 24]. To address this, we studied functional and molecular changes in ENS of A30P mice before onset of PD symptoms to identify gut-related biomarkers for early stages of PD.

## Methods

### Animals

α-synuclein-overexpressing transgenic A30P mice (Thy-1-SNCA-A30P [25]), based on a genetic C57B6/J background and age- and sex-matched wild type (WT) mice (postnatal day 2, adult 2 months and 12–13 months old) were used in experiments. Two-month-old A30P mice were defined as pre-symptomatic (ps)A30P mice and were used in expression profiling experiments. Animals were housed under specific pathogen-free conditions on a 12 h light/12 h dark cycle according to german regulations. For tissue dissections, adult mice were deeply anesthetized with isoflurane (Piramal Critical Care) and sacrificed by inhalation of an overdose. Newborn mice were killed by decapitation. Animals were dissected according to the guidelines of the local ethics committee and in accordance with the animal protection laws in Rhineland-Palatinate, Germany.

### Gait analysis

Gait was assessed in adult A30P and WT mice (2 months and 12–13 months old) using the Noldus CatWalk XT system. The system consists of a 70-cm long green illuminated glass runway. Above this corridor is a lid with red LED lights to create a silhouette of the mouse running in the corridor. Below the walkway, a high-speed camera records the scattered light from the paw prints, which is digitized and analyzed by CatWalk XT software. The calibrated walkway was set to a 10 × 20 cm area. Before the behavior test, mice were weighed. Mice were allowed to walk in an unforced manner on the glass plate at least five times in each experiment after a minimum training period of five runs the day before the experimental run. Mice that failed the training were excluded from the study. Footprints were classified automatically as right forepaw, right hindpaw, left forepaw, and left hindpaw. For each experiment, correct paw labels and footfall patterns were controlled, followed by an automated analysis of wide-range parameters involving spatial and temporal characteristics. After the gait analysis, the small intestine (SI) and the large intestine (LI) of 2-month-old and 12–13-month-old A30P and WT mice were removed and the gut lengths were measured.

### Tissue preparation

SI and/or LI segments from psA30P and WT mice (2 months, 12–13 months old and/or newborn) were collected in MEM-Hepes + 100 U/ml pencillin/100 μg/ml streptomycin (P/S) and the mesentery was removed. For full-thickness intestinal wall and whole-muscle layer preparations from adult mice, the segments were flushed with MEM-Hepes + 1% P/S to remove feces, followed by muscle layer dissection. Parts of the intestines were used to isolate the myenteric plexus (MP) for further studies. Brains were removed completely and dissected and processed appropriately.

#### Isolation of the MP

Primary enteric cells, in particular MP from newborn and adult mice, were isolated as previously described [26, 27]. In brief, stripped muscle layers were cut into small pieces and digested with 0.375 mg/ml Liberase (Roche) and 0.2 mg/ml DNase (Roche) in Hank’s balanced salt solution. Adult SI was incubated at 37 °C for 4 h and adult LI for 4.5 h. Thereafter, purified MP networks were washed two times with 0.01 M phosphate-buffered saline (PBS), frozen in liquid nitrogen, and stored at − 80 °C for further experiments. Tissue from newborn C57B6/J mice was digested for 2.5 h and MP cells were dissociated for cell culture experiments by incubation with TrypLE (Gibco) (2 × 6 min at 37 °C) and triturated into a single-cell suspension with 23G and 27G needles.

#### Isolation of the mesencephalon and the striatum

The mesencephalon was also isolated from psA30P and WT mice along with the MP. The brain was separated from the skin and the skull, and placed dorsal side up. The mesencephalon was dissected using a scalpel blade. A first cut was made adjacent to the inferior colliculi, followed by a second cut of approximately 4 mm in a rostral direction. The slice was placed with the rostral side up and the mesencephalon was taken out without including the hippocampus, cortex, or cerebellum. To dissect the striatum, the whole brain was sliced frontally in 2 mm sections, using a carbon steel razor blade. From the section where the striatum was easily to identify, the striatum was dissected with a fine tipped microscalpel. Both, the mesencephalon and the striatum were snap-frozen with liquid nitrogen and stored at − 80 °C for further experiments.

#### Whole-mount preparations

Segments of full-thickness intestinal wall and the outer muscle layer of psA30P and WT mice were stretched flat and fixed on a Sylgard plate followed by fixation with 4% paraformaldehyde for 2 h at 4 °C. Afterwards, samples were washed three times with PBS for 10 min. Intestinal wall samples were cut into 1 cm pieces, the muscle layers were removed, and samples were stored in 0.1% NaN_3_ in PBS at 4 °C for further experiments.

### Dopamine ELISA

Total dopamine levels in the striatum of A30P and C57B6/J mice (2 months and 12–13 months old) were quantified using a chemiluminescent enzyme-linked immunosorbent assay (ELISA) dopamine kit (enzolifesciences) according to the manufacturer’s instructions. 50 μl of each diluted (1:10 in 0.01 M PBS) homogenized sample was used for the ELISA analysis. Based on a standard curve absolute dopamine values were determined, while only samples within the detection range were used. To obtain the final dopamine content in the sample, the used tissue and buffer volumes were factored in as dilutions.

### Paraffin immunofluorescence (IF-P)

For Paraffin Immunofluorescence the gut tissues were fixed overnight with 4% formalin, dehydrated in a graded rising alcohol series (70, 80, 90 and 100%, each 2x3min) and embedded in paraffin wax. The paraffin embedded tissues were cut into 5 μm slides (cross sections) and stored at 4 °C.

Paraffin sections were deparaffinized in xylene substitute (3x10min), and a graded series of descending alcohol (100, 90, 80 and 70%, each 2x3min), followed by distilled water (2x3min) and TBST (5 min, 0.025% Triton-X-100 in TBS). For antigen retrieval sections were incubated with 0.01 M citric acid (pH = 6.1) for 30 min using a steamer. Cooled down sections were rinsed twice in TBST (0.025% Triton-X-100 in TBS) for 5 min and blocked in 10% normal serum with 1% BSA in TBS for 2 h at RT. Then primary antibodies (Supplementary Table 1), diluted in TBS with 1% BSA, were applied and incubated overnight at 4 °C. Again, samples were rinsed in TBST (0.025% Triton-X-100 in TBS) for 5 min before the secondary antibodies (Supplementary Table 1), diluted in TBS with 1% BSA, were added and incubated for 1 h at RT. After washing three times for 5 min in TBS sections were mounted with fluorescent mounting medium.

### GI motility recordings

Video recording with spatiotemporal mapping is a reliable technique for monitoring and displaying motility of gut segments. To evaluate GI motility in psA30P and WT mice, a luminal perfusion setup was used as previously described by Schreiber et al. 2014 [28]. In brief, a 3.5-cm gut segment of the ileum and proximal LI were dissected and the mesentery was removed. Then, the tissue was fixed on Luer locks in an organ bath filled with tyrode buffer (130 mM NaCl, 24.2 mM NaHCO_3_, 11 mM glucose, 4.5 mM KCl, 2.2 mM CaCl_2_, 1.2 mM NaH_2_PO_4_, 0.6 mM MgCl_2_), gassed with carbogen to reach a stable physiological pH 7.4. The perfusion bath was kept at 37 °C using a heat exchanger. Intestinal segments were luminally perfused at a flow rate of 0.2 ml/min while the height of the luminal efflux tubing relative to the water level (3 cm) was set to a corresponding luminal pressure. All preparations were allowed to equilibrate for 10 min followed by a 10 min spontaneous motility period.

GI motility was assessed by video recordings at 25 frames/s. Videos were analyzed and quantified by MotMap (www.smoothmap.org) and a custom written LabVIEW program (LabVIEW 2019, National Instruments). The video analysis software MotMap was used to set 30 imaginary dots along a 2 cm gut segment on the lower and upper border of the gut as seen in Fig. 3a. The up and down movements of these dot pairs were tracked automatically to calculate the positions to each other for every frame, representing the amplitude of gut activity (∆). Before the analysis, the program automatically applies a low frequency baseline correction to each of the 30 sections to compensate for slow gut movements. To validate amplitude changes, all values were normalized to their initial diameter. The motility data represent dilatations as positive values vs. the average diameter and contractions as negative values. Motility was converted to high-resolution spatiotemporal heatmaps, while all values were specified in a color range using the custom written LabVIEW software. Gut contractions were counted using spatiotemporal heatmaps. The mean interval was defined as the duration of one contraction and one dilatation (Fig. 3a) and indicates the corresponding frequency. The contraction duration was calculated as the time between adjacent leading edge to leading edge zero crossings. To suppress a noise-induced false triggering, a hysteresis threshold was set at the order of 0.05 to 0.1 mm. Each of the 30 dots over the 2 cm was separately analyzed by determining the duration of all full cycles followed by averaging over all detected cycle durations. To determine the velocity of GI motility, slopes of individual contractions were measured in the LabView program by manually aligning a cursor over the wave top, as shown in Fig. 3b. The program calculates the velocity from the start and end point of the cursor. Together, mean intervals, contraction numbers, and contraction velocity provide information about peristaltic activity.

### Protein isolation for mass spectroscopy of the MP

Protein profiles of purified MP from SI and LI tissue obtained from psA30P and WT mice were investigated by mass spectroscopy. Harvested MP networks were thawed and transferred into 0.1 μM PBS, containing 1:100 protease inhibitor (Roche) and nuclease mix (GE Healthcare), and were vortexed. After five freeze-thaw cycles in liquid nitrogen, samples were purified with the 2-D Clean up kit (GE Healthcare). Pelleted proteins were resuspended in 6 M urea, 2 M thiourea and 25 mM NH_4_HCO_3_ at pH 8. The protein concentration was measured using the 2-D Quant kit (GE Healthcare). A total protein amount of approximately 16 μg for the colon and 25 μg for the small intestine was determined.

Mass spectrometry analysis was performed on a high-resolution LC-MS system (Eksigent nanoLC 425 coupled to a Triple-TOF 6600, AB Sciex) in information-dependent acquisition (IDA) mode. HPLC separation was performed in trap-elution mode using a Symmetry C18 column (5 μm particles, 0.18 × 20 mm, Waters) for trapping and a self-packed analytical column (75 μm × 150 mm, 3 μm particles ReproSil-PurC18-AQ) for separation. A constant flow of 300 nl/min was used and the gradient was ramped within 108 min from 2 to 33% of HPLC buffer B (buffer A: 2% acetonitrile, 0.1% formic acid; buffer B: 90% acetonitrile, 0.1% formic acid), then within 12 min to 50% buffer B, followed by washing and equilibration steps. The mass spectrometer was run in IDA mode and recorded one survey scan (250 ms, 350–1500 m/z) and fragment spectra (70 ms, 100–1400 m/z) of the 25 most intense parent ions (charge state > 2, intensity > 300 cps, exclusion for 15 s after one occurrence), giving a cycle time of 2 s. In total, 1044 different proteins were investigated (Supplementary Table 2).

In the mass spectrometry-based proteomics workflow followed here, the quantification of proteins depends on the integration of quantitative information from several independent tryptic peptides, which is derived from extracted ion chromatograms (XICs) obtained for each peptide at the MS1 level. The quantitative information derived is very reliable and more robust compared with western blotting. We have demonstrated this recently by comparing MS-based quantification with quantification by western blots, where quantified changes were the same, but MS-based quantification was associated with less variance [29]. Hence, using a more inaccurate method to validate a more accurate one seems counterintuitive.

The yielded proteins were then analyzed and visualized by Search Tool for the Retrieval of Interacting Genes/Proteins (STRING) and Ingenuity Pathway Analysis (IPA) software tool (QIAGEN Inc., https://www.qiagenbioinformatics.com/products/ingenuitypathway-analysis).

### Whole-mount immunostaining

Immunostaining and clearing procedures for the full-thickness intestinal wall and muscle layer samples were performed as previously described [30]. In brief, whole-mount muscle layer and intestinal wall preparations of LI specimens from psA30P mice and corresponding WT were permeabilized for 4 h at 37 °C on a shaker with permeabilization solution (0.01% NaN_3_ + 1% normal donkey serum + 1% Triton-X-100 in PBS). Then, samples were blocked in blocking solution (0.01% NaN_3_ + 10% normal donkey serum + 0.1% BSA + 1% Triton-X-100 in PBS) for 4 h at room temperature (RT) on a shaker. Subsequently, samples were incubated with primary antibodies (diluted in blocking solution, Supplementary Table 1) at 37 °C for 48 h using an orbital shaker. After rinsing four times in PBST (0.05% Tween20 in PBS) for 30 min, tissues were incubated with respective secondary antibodies (1:500 in 0,01% NaN_3_^+^ 1% Triton-X-100 in PBS) overnight at 37 °C on an orbital shaker (Supplementary Table 1). Again, samples were rinsed with PBST four times for 30 min and then counterstained with DAPI (1:1000) for 2 h at RT. Samples were washed in PBS five times for 10 min at RT. For the clearing procedure, Histodenz (Sigma) was diluted with melted N-methylacetamide (40% in PBS, Sigma) to 86% (w/v) concentration and incubated at 37 °C until dissolved. Then 0.1% Triton-X-100 and 0.5% 1-thioglycerol were added to the clearing solution. After being incubated in completed clearing solution for 1–5 min, the outer muscle layers were mounted in fluorescent mounting medium. Detection and image processing were performed with CellObserver Z1 using the ApoTome technology and Axiovision software (Zeiss). The quantification of the immunofluorescence images was performed using ImageJ software. To quantify the expression per picture section, fluorescence images were converted to binary pictures, where the immunostained areas were presented in white and the percentage of positive area per picture section was calculated.

Full-thickness samples were incubated in completed clearing solution overnight and imaged on a Leica SP8 confocal microscope (Leica Microsystems CMS GmbH, Germany). For imaging, samples were positioned in a glass-bottomed petri dish, submerged in the clearing solution and covered by a golden ring with nylon mesh to prevent the tissue from floating. 3-D renderings from intestinal wall samples were obtained using the 3-D Viewer of the Leica LAS X software (Leica microsystems, Wetzlar, Germany).

### In vitro studies

#### Aggregation of A30P α-synuclein

The A30P α-synuclein (kindly provided by Hilal Lashuel) was purified by size exclusion chromatography followed by reverse phase chromatography, which ensures removal of endotoxins. To create aggregated α-synuclein peptides, 50 μM of endotoxin free A30P α-synuclein and 50 μM dopamine (Sigma) were diluted in 5 mM Tris-HCl with 100 mM NaCl at pH 7.4 using low protein binding tubes. The aggregation process was enforced by permanent shaking for 3 days at 37 °C as previously described [31].

#### Cell treatment with A30P α-synuclein

Dissociated MP cells from newborn mice were plated on extracellular matrix (ECM) (Sigma)-coated coverslips (40.000 cells/coverslip). Cells were maintained for 7 days in differentiation medium consisting of DMEM/F-12 medium, 1% bovine serum albumin (BSA), 2% B27 with retinoic acid supplement, 0.1% β-mercaptoethanol, P/S and 10 ng/μl human glial cell-derived neurotrophic factor (Immuno Tools) before exposure to monomeric and aggregated A30P α-synuclein. Culture medium was replaced by medium with B27 without antioxidants, containing 0.5 μM A30P α-synuclein in Tris-HCl. After α-synuclein treatment for an additional 5 days, cells were fixed with 4% formaldehyde for 15 min at RT and were washed 3 times with 0.01 M PBS. To determine cell viability after α-synuclein treatment, a live-dead-assay was performed. Cells were incubated with 1 μg/ml calcein and 0.5 μg/ml propidiumiodide in 0.01 M PBS for 15 min at 37 °C and analyzed with the CellObserver Z1 and Axiovision software (Zeiss). Total numbers of living and dead cells were calculated per picture section.

#### Immunocytochemistry

Cells were fixed with 4% formaldehyde and permeabilized with 0.1% Triton-X-100 for 10 min, followed by 30 min blocking in 0.01 M PBS with 10% normal donkey serum. Primary antibody incubation (Supplementary Table 1) was performed for 1 h at RT in 0.01 M PBS. Samples were washed three times in PBS and further incubated with respective secondary antibodies (1:500, Supplementary Table 1) in 0.01 M PBS for 1 h at RT. Samples were washed three times in PBS and once in distilled water. Cells were then mounted on slides with fluorescent mounting medium with DAPI. Detection and image processing were performed with CellObserver Z1 using the ApoTome technology and Axiovision software (Zeiss). To determine the ratios of Nefl and Calb2 positive cells to the total number of neurons, numbers of cells positive for each protein were manually counted and divided by the number of PGP9.5 positive neurons.

### nCounter analysis

For miRNA profiling, total RNA (including small RNAs) from the MP of the LI and the mesencephalon samples from psA30P and WT mice was isolated with the miRNeasy Micro Kit (Qiagen). RNA was eluted in 10 μl (50–100 ng/μl) nuclease-free H_2_O. RNA quantity and quality were assessed using the Qubit™ 4 Fluorometer (Qubit RNA HS Kit; Invitrogen) and the NanoDrop 2000 (Thermo Fisher Scientific). Agilent Bioanalyzer 2100 (TotalRNA Nanokit) and miRNA profiling was performed using the nCounter SPRINT system (NanoString Technologies, USA, www.nanostring.com) at the nCounter Core Facility of the University of Heidelberg (Heidelberg, Germany, www.ncounter.uni-hd.de). For miRNA library preparation and subsequent probe hybridization using the mouse miRNA panel 1.5 (Supplementary Table 3), 100 ng of total RNA was applied. Using the standard nCounter miRNA assay protocol, expression profiles of 578 mouse miRNAs derived from miRbase v.22 were determined. Only miRNAs with at least 100 counts were considered as robustly expressed and included in differential expression analysis of psA30P vs. WT tissue. Data were analyzed using the nSolver software version 4.0 (NanoString Technologies). Stably expressed housekeeping genes such as beta-actin (*Actb*), beta-2-microglobulin (*B2m*), glyceraldehyde 3-phosphate dehydrogenase (*Gapdh*), and ribosomal protein L19 (*Rpl19*) were used for data normalization. To investigate a potential impact of the dysregulated miRNAs on target gene translation, the top-regulated miRNAs were subjected to target gene prediction using mirWALK 3.0 integrating the algorithms of multiple miRNA target prediction tools [32].

To gain further insight into the function of the most dysregulated miRNAs and their targets, data were put into biological context using the knowledge-based IPA. IPA integrates selected data sets (in our case, miRNA profiles and proteomics) with mining techniques to predict functional connections, protein networks, protein-protein interactions, and related biological functions as well as canonical signaling pathways. miRNA target filter plus miRNA/target = protein pairing analysis were used to identify co-regulated miRNA/target mRNA pairs.

### Statistical analyses

For statistical analysis, GraphPad Prism Software 8 was used. Normality of the sample populations was tested with the Shapiro-Wilk test. For normally distributed data, group differences were analyzed with the Student’s t-test and the associated effect sizes with the Cohen’s test [33] or two-way ANOVA. Data are displayed as means ± SD and *p*-values ≤ 0.05 (*), ≤ 0.01 (**) and ≤ 0.001 (***) were considered statistically significant.

## Results

### 2-month-old A30P mice are pre-symptomatic (psA30P mice)

**Fig. 1:**
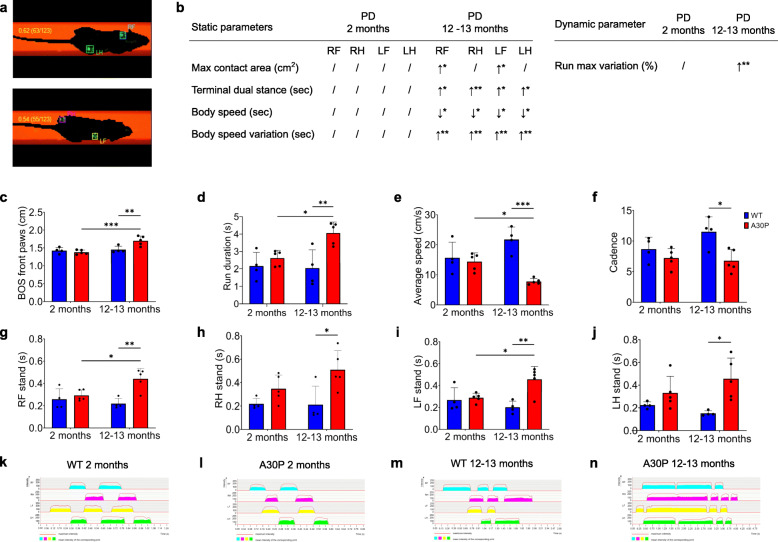
CatWalk XT analysis of different aged A30P and wild type (WT) mice. (**a**) Individual paws were classified automatically with the CatWalk XT software as right forepaw (RF), right hindpaw (RH), left forepaw (LF), and left hindpaw (LH). (**b**) Static and dynamic parameters were all significantly different in older A30P mice compared with age-matched controls, while 2-month-old A30P and WT mice did not show any motoric disturbances. Base of support on front paws (**c**) and run duration (**d**) were significantly increased in 12–13-month-old A30P mice, while the average speed (**e**) and cadence (**f**) were decreased. Stands of all paws, including RF (**g**), RH (**h**), LF (**i**), and LH (**j**), were significantly augmented in 12–13-month-old A30P mice compared with WT animals. This was confirmed by corresponding 2-D maps from WT (**k**, **m**) and A30P mice (**l**, **n**). Two-month-old A30P mice showed no motoric changes, which was confirmed by 2-D maps. WT mice are shown in blue (*n* = 4), A30P mice in red (*n* = 5). 2-D maps determine the gait sequence of paw contact with the glass plate over time. * *p* ≤ 0.05, ** *p* ≤ 0.01, and *** *p* ≤ 0.001 using ANOVA

Gait analysis has been used to show onset of obvious PD manifestations in mice [34]. We analyzed ten gait parameters of A30P and wild type (WT) mice using the CatWalk XT system. Animal footprints were automatically captured by the video camera of the system and were further categorized into individual paws like right forepaw (RF), right hindpaw (RH), left forepaw (LF), and left hindpaw (LH) via the software (Fig. 1a). To characterize pre-symptomatic PD or phenotype-manifested PD in mice, we assessed the gait in two age groups: 2 months old and 12–13 months old. Body mass was not different between 2-month-old A30P and WT mice, but 12–13-month-old A30P mice were significantly heavier than age-matched WT mice (Supplementary Fig. 1). As expected, the older A30P mice showed significant motor impairments in all tested parameters compared to age-matched controls: maximum contact area, terminal dual stance, body speed, body speed variation, run maximum variation (*p* ≤ 0.05, Fig. 1b and Supplementary Fig. 2), base of support on front paws (Fig. 1c), run duration (Fig. 1d), average speed (Fig. 1e) and cadence (Fig. 1f). In addition, the older mouse group displayed significantly extended stands (*p* ≤ 0.05, Fig. 1g–j) and markedly changed footprint patterns from all paws (Fig. 1k-n). In contrast, younger A30P mice showed no significant changes in all investigated gait parameters compared with age-matched WT mice, indicating that these mice have a pre-symptomatic stage of the disease. We also measured gut length in these mice. Gut length did not differ between A30P and WT mice in both age groups, but both intestines were significantly longer in 12–13-month-old A30P and WT mice compared with 2-month-old A30P and WT mice (Supplementary Fig. 3).

**Fig. 2:**
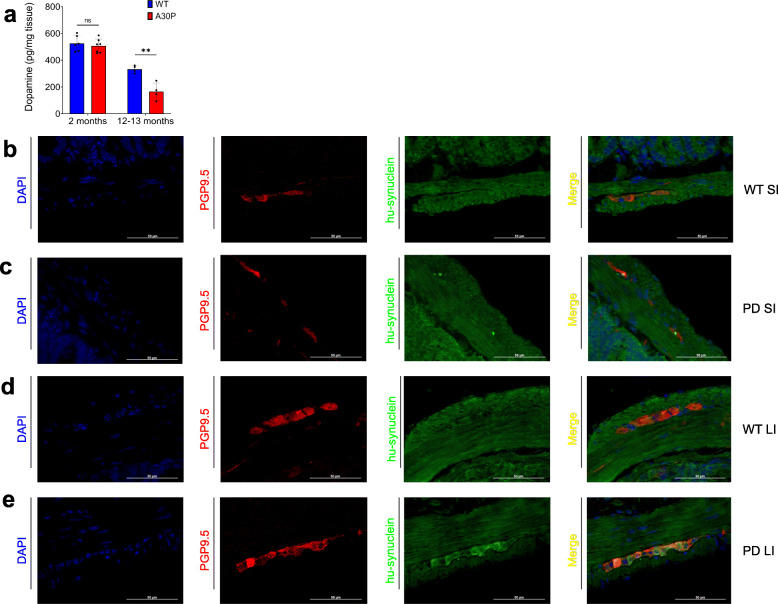
Striatal dopamine levels and intestinal synuclein aggregates in A30P and wild type (WT) control mice. (**a**) The amount of dopamine in the striatum of 2- and 12–13-month-old A30P and WT mice was determined by an enzyme-linked immunosorbent assay (ELISA). In the younger group (2 months) no changes were found in the striatal dopamine content, while the older A30P group exhibited significantly lower dopamine levels compared with their age-matched controls mice. WT animals are shown in blue, A30P mice in red. Quantitative data are expressed by means ± SD from segments of *n* = 6 for 2-month-old and n = 4 for 12–13-month-old PD and WT mice. ** *p* < 0,01 vs. wild type and control respectively by Student’s t test and Cohen’s d (Supplementary Fig. 7a); ns = not significant. Detection of overexpressed aggregated human α-synuclein (hu-synuclein) in the gut of 2-month-old mice revealed that there were no aggregates present in both intestines of the WT mice (**b**, **d**), while several aggregates were found in the small (**c**) and the large intestine (**e**) of the A30P mice. For synuclein detection n = 5 for A30P and WT were investigated

To proof that 2-month-old A30P mice do not show a significant PD pathology in the brain yet, we assessed striatal dopamine levels of 2-month-old and 12–13-month-old A30P and WT mice. Therefore, we isolated the striatum of these mice and measured the dopamine content using an ELISA (Fig. 2a). In the suspected pre-symptomatic PD group (2 months old) we found no significantly altered dopamine levels (475,23 pg ± 100,78) compared with the corresponding WT (525,67 pg ± 57,27), revealing no significant brain pathology at this time point. The older A30P group instead, which also shows motoric impairments in the CatWalk, displays significantly reduced striatal dopamine levels (165,40 pg ± 64,65; *p* ≤ 0.01) compared with the age-matched WT group (332,38 pg ± 27,46) indicating a diseased brain.

But to give evidence for a pre-manifestation of PD in the gut in the 2-month-old mice, we identified disease causing overexpressed α-synuclein aggregates (human A30P α-synuclein) in the small and the large intestine of both, 2-month-old PD and WT mice, using Paraffin immunofluorescence. We were able to detect overexpressed human α-synuclein aggregates in the SI (Fig. 2c) as well as in the LI (Fig. 2e) of A30P mice. Interestingly, the expression level is less prominent in the SI, while in the WT mice no human α-synuclein could be found in both intestines (Fig. 2b, d).

Based on these findings, the non-motor-impaired 2-month-old PD group with intestinal but no brain α-synuclein pathology, was defined as the pre-symptomatic A30P mouse group (psA30P), and was used for further investigations.

### Altered GI motility in psA30P mice

**Fig. 3:**
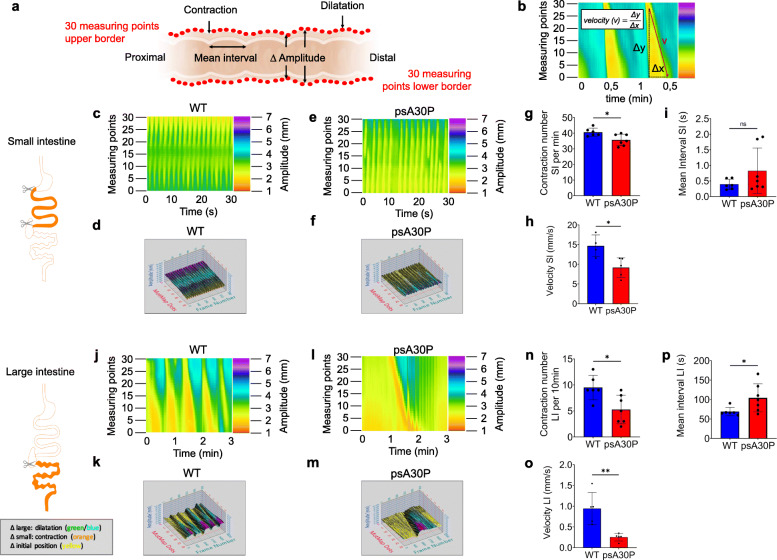
Motility patterns during ex vivo luminal perfusion experiments in pre-symptomatic (ps)A30P mice and wild type (WT) controls. (**a**) Video recordings of gut movements were analyzed with MotMap and LabView to determine alterations in amplitudes **∆**, mean intervals and contraction numbers. (**b**) Schematic illustration of the velocity measured by LabView. Spatiotemporal maps and corresponding 3-D MotMaps showed altered small intestine (SI) motility (**c**–**f**) and large intestine (LI) motility (**j**–**m**). Less SI and LI motility was shown in psA30P mice compared with WT. The gut diameter in the heatmaps (pixel color) is indicated on the y-axis, the time on the x-axis. Contraction numbers were significantly lower in the SI (**g**) and the LI (**n**) of psA30P mice compared with WT controls. Mean intervals were slightly higher with more variation in the SI of psA30P mice compared with WT (**i**), while in the LI of psA30P mice mean intervals were significantly prolonged compared with WT (**p**). Velocity was significantly decreased in the SI and LI of psA30P mice compared with WT (**h**, **o**). WT mice are shown in blue, psA30P mice in red. Calculations were performed with LabView and GraphPad Prism 8. Quantitative data are expressed as means ± SD from segments of *n* = 7 psA30P mice and *n* = 6 age-matched WT controls for mean interval and contraction numbers. For velocity determination, *n* = 5 psA30P mice and n = 5 WT mice were examined. * *p* ≤ 0.05, and ** *p* ≤ 0.01 using Student’s t test and Cohen’s d (Supplementary Table 7b), ns = not significant

To investigate the hypothesis that the gut is the first site of PD manifestation, we compared GI motility patterns in the SI and the LI of psA30P mice and age-matched WT animals in isolated gut segments. We measured GI movement using several parameters, including changes in amplitude (∆ amplitude), number of contractions, mean interval (Fig. 3a), and velocity of motions (Fig. 3b).

Both, the SI and the LI, had an isochronous motility pattern in WT mice (SI: Fig. 3c,d; LI: Fig. 3j,k), whereas psA30P mice showed a discontinuous outline with extended passive interphases (SI: Fig. 3e,f; LI: Fig. 3l,m).

In the SI, more contractions were observed in WT mice (40.7 ± 2.6 contractions/min) than in psA30P mice (35.7 ± 3.6 contractions/min; *p* ≤ 0.05, Fig. 3g). Furthermore, the movements of WT mice were significantly faster (14.7 ± 2.8 mm/s) than those of psA30P mice (9.2 ± 2.2 mm/s; *p* ≤ 0.05, Fig. 3h). The mean interval was higher in psA30P mice because of extended and variable interphases of (0.50 ± 0.1 s) compared with WT mice (0.39 ± 0.7 s), but this difference was not significant (Fig. 3i).

In the LI, WT mice exhibited 9.5 ± 2.4 contractions (Fig. 3n) with an appropriate velocity of 0.9 ± 0.4 mm/s (Fig. 3o) over a 10-min period. The number (5.3 ± 2.6 contractions per 10 min; *p* ≤ 0.05, Fig. 3n) and velocity of contractions (0.3 ± 0.1 mm/s; *p* ≤ 0.01, Fig. 3o) were significantly lower in psA30P mice. In addition, the mean interval was significantly shorter in WT mice (69.1 ± 10.8 s) compared with in psA30P mice (104.4 ± 33.4 s; *p* ≤ 0.05, Fig. 3p).

The reduced gut motility in psA30P mice can be visualized in the video recordings (Supplementary Videos 1, 2, 3, 4); these videos show retarded activity, slower contractions, and longer static phases in psA30P mice compared with WT mice (Fig. 3).

### Dysregulated protein expressions in the ENS of psA30P mice

To investigate the role of the ENS in PD pathogenesis, we dissected the myenteric plexus (MP) of the SI and the LI in psA30P and WT mice. Whole protein was isolated from the gut tissue, separated by high-performance liquid chromatography (HPLC), and further analyzed by mass spectroscopy.

**Fig. 4:**
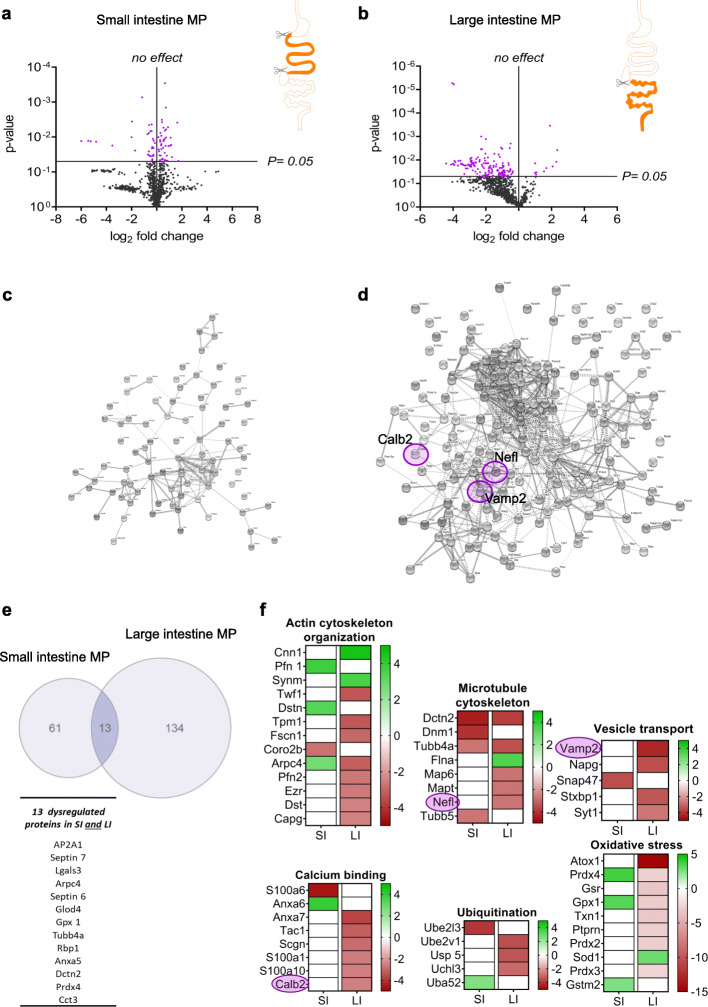
Mass spectroscopy of the myenteric plexus (MP) from pre-symptomatic (ps)A30P mice compared with wild type (WT) controls. Regulated proteins in the MP of the small intestine (SI, **a**) and the large intestine (LI, **b**) were volcano plotted using GraphPad Prism 8 according to their statistical significance. Proteins with significantly altered expression are shown in purple, proteins with non-significantly altered expression in black. Protein-protein interactions obtained from the STRING database are shown for MP samples of the SI (**c**) and the LI (**d**). Neurofilament light chain (Nefl), vesicle-associated membrane protein 2 (Vamp2), and calbindin 2 (Calb2) are highlighted with purple circles. (**e**) In total, 74 proteins were affected in the SI and 147 in the LI. Among these, 13 proteins were changed significantly in both, the SI and the LI. The Venn diagram and the heatmaps were generated using GraphPad Prism 8. Here, only proteins with significantly altered expression were included. Classified functional groups of regulated proteins in the gut (actin and microtubule organization, vesicle transport, calcium binding, ubiquitination, and oxidative stress) are shown in (**f**). The intensity levels (percentage of normalized volumes) of the protein spots were shown on a heatmap according to their expression levels. Individual rows represent single spots and graduated scale color codes from red (decreased expression levels) to green (increased expression levels) and white (no altered expression levels). Each column represents data from n = 4 independent experiments

The MP protein expression profile was different between psA30P and corresponding WT mice in the SI (Fig. 4a) and LI (Fig. 4b). In total, 1044 proteins were detected by mass spectroscopy; in 7% of proteins, expression was significantly altered in the SI (Fig. 4a, Supplementary Fig. 4a, Supplementary Table 2) and almost twice as many proteins (14%) in the LI (Fig. 4b, Supplementary Fig. 4b and Supplementary Table 2), indicating a stronger and maybe even an earlier manifestation of PD at this anatomical site. Proteins with significant changes in expression were analyzed and visualized by Search Tool for the Retrieval of Interacting Genes/Proteins (STRING). Each dot represents a single protein, while clustered proteins or proteins in the direct neighborhood indicate a similar function (Fig. 4c and d; Supplementary Fig. 4).

We used a Venn diagram to illustrate the distribution of dysregulated proteins in the SI (74 proteins) and the LI (147 proteins); 13 proteins were differentially expressed in both tissues (Fig. 4e). Categorization into groups by the STRING database yielded i.a. six different functional clusters: actin and microtubule organization, vesicle transport, calcium binding, ubiquitination, and response to oxidative stress (Fig. 4f). Proteins in these groups are known to be involved in PD pathogenesis in mice and humans, mainly affecting the CNS [35–55]. This functional classification emphasized that more proteins are dysregulated in the LI than in the SI. Therefore, we focused all further investigations on the LI. We selected three proteins with altered expression for detailed examinations: neurofilament light chain (Nefl), vesicle-associated membrane protein 2 (Vamp2) and calbindin 2 (Calb2). We chose these proteins because they have already been implicated in PD pathology. For example, Nefl [56–58] and Calb2 [59–62] expression is altered in the brain of humans with PD as well as in PD models, but nothing is known about how these proteins are affected in the gut during early stages of PD. In addition, both, Nefl and Calb2 can co-localize with α-synuclein [63, 64]. Several studies have shown a role for Vamp2 in neurodegeneration [44, 46, 48, 65, 66], but no direct link to PD pathogenesis has been described to date, although Vamp2 does bind to α-synuclein [67].

### Differential Nefl, Calb2, and Vamp2 expression in the ENS of psA30P mice

**Fig. 5:**
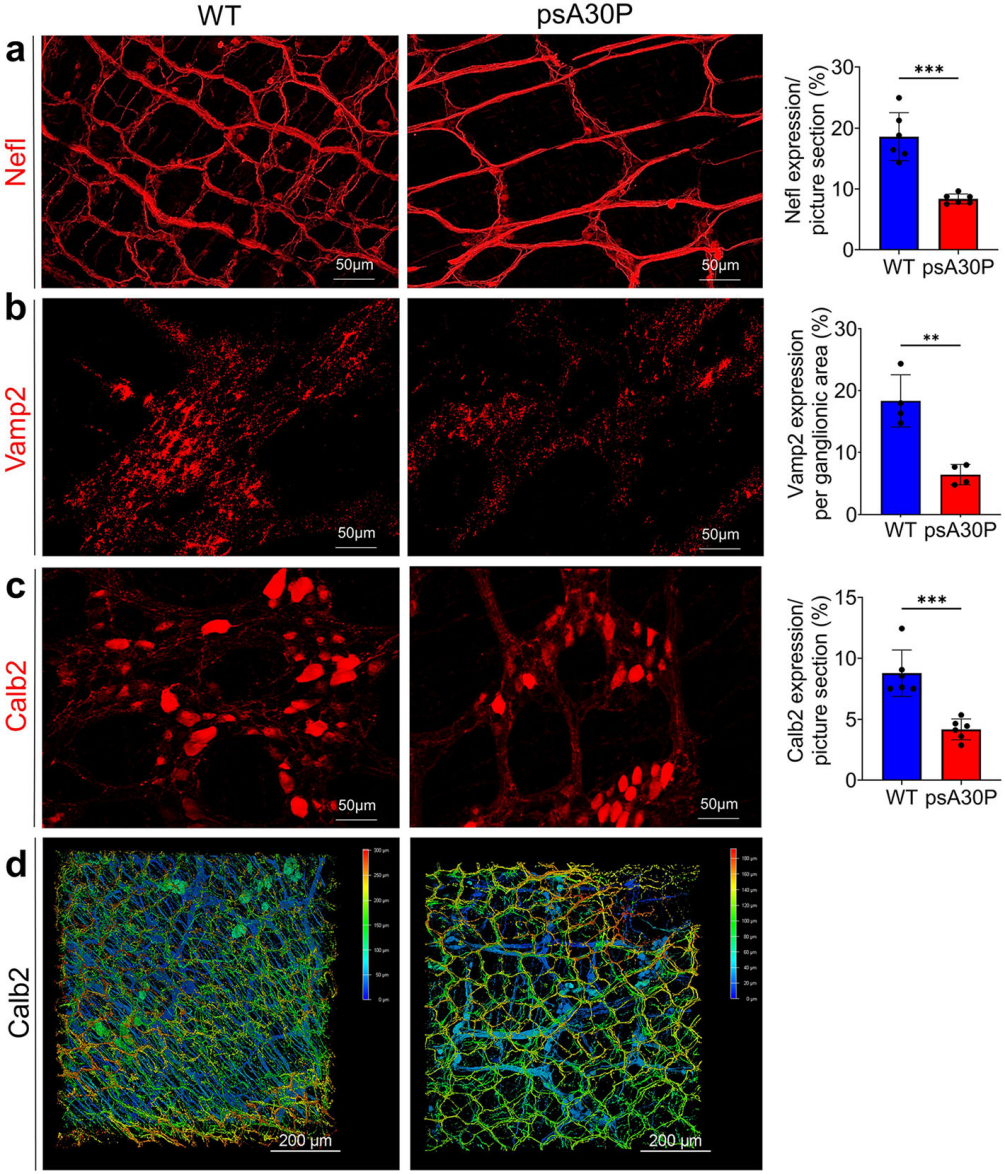
Effect of A30P α-synuclein on neuronal markers in whole-mounts muscle layers from pre-symptomatic (ps) A30P mice. Distribution of neurofilament light chain (Nefl) (**a**), vesicle-associated membrane protein 2 (Vamp2) (**b**) and calbindin 2 (Calb2) (**c**), in full thickness muscle layers of the large intestine (LI) in psA30P and wild type (WT) mice. There were significantly fewer Nefl-, Vamp2-, and Calb2-positive cells in pre-symptomatic (ps)A30P mice than in WT mice. Three-dimensional (3-D) images were made of full thickness intestinal walls immunostained for Calb2 (**d**) and confirmed the 2-D impressions (**c**) of muscle layer stainings. WT mice are shown in blue, psA30P mice in red. Quantitative data were analyzed with ImageJ and are expressed as means ± SD from n = 5 independent experiments using GraphPad Prism 8. Nefl and Calb2 expressions were calculated per picture section, Vamp2 expression was analyzed per ganglionic area (class III beta tubulin (Tuj1) positive area). ** *p* ≤ 0.01 and *** *p* ≤ 0.001 using Student’s t test and Cohen’s d (Supplementary Table 7d). 3-D images are presented using the depth-coding mode, where a depth color code corresponds to the position within the volume. The scale bar displays the spatial distribution of the color coding. The serosal side of whole-tissue samples was set at 0 μm and is depicted in blue. The top of the villi is displayed in red. Images are shown as a top view

To evaluate the expression of Nefl, Calb2, and Vamp2 in the LI during pre-symptomatic early-onset PD, we performed immunohistochemical stains on whole-mounts of the LI from psA30P and WT mice (Fig. 5). Based on our proteomic data and because of their pivotal role in the ENS, we decided to see whether these proteins can serve as biomarkers for early PD. Preliminary assessments of the LI whole-mounts showed a significantly smaller ganglionic area in psA30P mice than in WT mice (Supplementary Fig. 5).

Nefl staining revealed a large number of myenteric ganglia that were evenly distributed over the underlying circular muscle layer. Nefl-positive cells were also frequently encountered in the interconnecting strands running parallel to the circular muscle layer. These fibers had a thinner and smoother morphology than those running longitudinally. In general, Nefl expression was weaker in psA30P mice (8.4 ± 0.8%) than in WT mice (18.6 ± 3.9%; *p* ≤ 0.001, Fig. 5a). Fiber density was especially reduced in the interconnecting strands. Consistently, there were significantly fewer synaptic vesicles in the muscle layers of psA30P mice (6.4 ± 1.6%) compared with WT animals (18.3 ± 4.2%; *p* ≤ 0.01, Fig. 5b). Calb2 showed a prominent immunoreactivity in the cytoplasm of neuronal soma and in axonal projections in both, psA30P and WT mice. However, there was a significantly higher Calb2 expression in WT mice (8.8 ± 1.5%) than in psA30P mice (4.2 ± 0.86%; *p* ≤ 0.001, Fig. 5c). Calb2 is expressed not only in the MP, but also in the submucosal plexus, which is located in the submucosal layer. Therefore, we imaged the whole gut in 3-D to include both plexuses, and confirmed the reduced Calb2 expression in psA30P mice (Fig. 5d).

### Differential Nefl, Calb2, and Vamp2 expression in primary ENS cells from an in vitro PD model

**Fig. 6:**
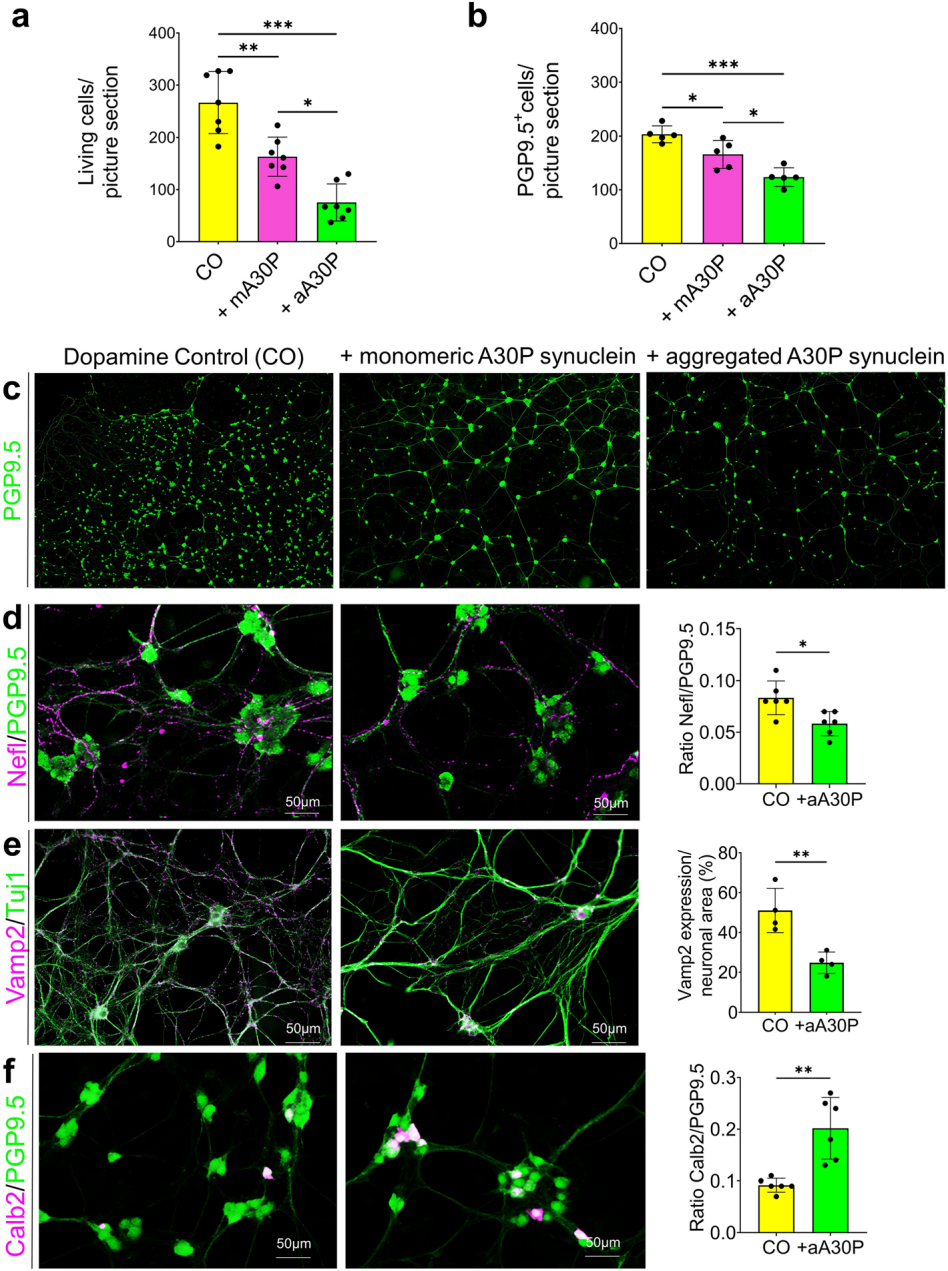
Effect of A30P α-synuclein on enteric cells. For in vitro studies, myenteric plexus (MP) cells isolated from C57B6/J mice (postnatal day 2) were treated with 0.5 μM monomeric and aggregated A30P α-synuclein for 5 days in vitro. This significantly reduced the number of living cells (**a**) and protein gene product (PGP) 9.5-positive neurons (**b**, **c**), while the toxicity was more intense using the aggregated form. Addition of aggregated A30P α-synuclein to the MP cells resulted in a significant reduction of Nefl positive neurons (**d**) and significantly less synaptic vesicles (**e**) compared with the dopamine control, as well as in significantly more Calb2 positive neurons (**f**). For Nefl and Calb2 data are indicated as a ratio of Nefl and Calb2 to PGP9.5 normalized to control. Calculation of Vamp2 expressions was restricted to neuronal (class III beta tubulin (Tuj1) positive) areas. Quantitative data are expressed by means ± SD from five to seven independent experiments (*N* = 90 images per condition). Dopamine controls are shown in yellow, monomeric α-A30P synuclein treated cells in pink and aggregated α-A30P synuclein treated cells in green. * *p* < 0,05; ** *p* < 0,01 and *** *p* < 0,001 vs. dopamine control, respectively, by Student’s t test and Cohen’s d (Supplementary Table 7d) and ANOVA for grouped analyses

To verify the dose-dependent toxicity of the mutant A30P α-synuclein protein on primary MP cells, we performed a live-dead assay as a pilot experiment with different incubation times (not shown) using MP cells from postnatal C57B6/J mice which were treated with endotoxin-free monomeric and aggregated A30P α-synuclein. Based on these results, we chose a concentration of 0.5 μM for the A30P α-synuclein, and an incubation time of 5 days for further experiments. A30P α-synuclein administration lead to a significantly increased number of dead cells for both (Fig. 6a), the monomeric (163.20 cells ±37.54) and the aggregated form (75.32 cells ±35.52), while this reduction was much more severe when enteric cells were exposed to the aggregated form (reduction monomer: 38.83%, reduction aggregate: 71.77%) compared with the dopamine control (266.77 cells ±59.38) (Fig. 6a).

To quantify the total number of neurons after A30P α-synuclein treatment, cultivated cells were stained with the neuronal marker protein gene product (PGP) 9.5 (Fig. 6b,c). The amount of neurons was significantly reduced (18.43%) after addition of monomeric A30P α-synuclein (WT: 203.54 ± 15.60 PGP9.5-positive cells; monomeric A30P: 166.02 ± 26.06 PGP9.5-positive cells; *p* ≤ 0.05, Fig. 6b,c) and even more significantly decreased (39.23%) when exposed to the aggregated A30P α-synuclein form (123.69 ± 17.43 PGP9.5-positive cells; *p* ≤ 0.001, Fig. 6b,c). To further analyze the cellular effects of the more damaging aggregated form of A30P α-synuclein on the expression of neuronal subtypes and synaptogenesis, additional immunocytochemical stainings were performed for class III beta tubulin (Tuj1), Nefl, Calb2, and Vamp2. The same picture as for PGP9.5. appears with the Nefl stainings, where we found significantly fewer Nefl-positive cells (WT: 0.08 ± 0.02 ratio Nefl/PGP9.5; A30P: 0.06 ± 0.01 ratio Nefl/PGP9.5; *p* ≤ 0.05, Fig. 6d and Supplementary Fig. 6a). An examination of synaptic vesicles revealed significantly less Vamp2-positive signals for aggregated A30P α-synuclein-treated cells per neuronal area (WT: 53.7 ± 11.1%; A30P: 24.7 ± 5.4%; *p* ≤ 0.01, Fig. 6e). Conversely, we found nearly twice as many Calb2-positive neurons in α-synuclein-aggregate-treated cells compared with the unchallenged cultures (ratio Calb2/PGP9.5 WT: 0.09 ± 0.01; A30P: 0.2 ± 0.06, *p* ≤ 0.01, Fig. 6f and Supplementary Fig. 6b), which might reflect the described higher resistance of calbindinergic neurons to toxic impacts [68].

### psA30P mice exhibit increased oxidative stress

**Fig. 7:**
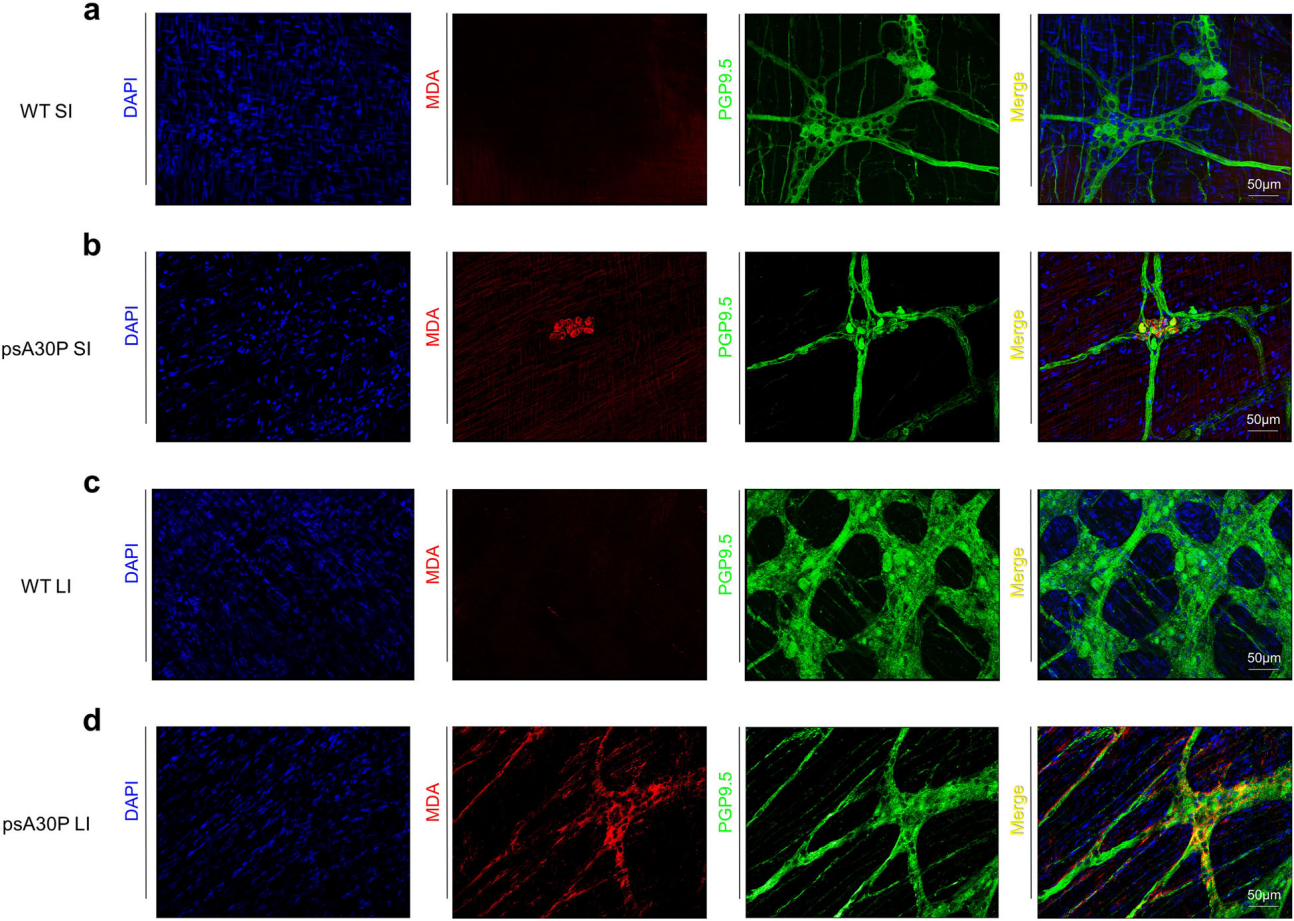
Analysis of oxidative stress in pre-symptomatic (ps) A30P mice. The effect of overexpressed human α-synuclein on lipid peroxidation was shown by whole-mount immunostainings of the small intestine (SI) and the large intestine (LI) from 2-month-old psA30P and wild type (WT) mice. Tissues were stained with malondialdehyde (MDA, red) used as an oxidative stress marker, with protein gene product (PGP, green) 9.5 as a neuronal cell marker and with 4′,6-Diamidin-2-phenylindol (DAPI, blue) for nuclear detection. In WT mice no oxidative stress levels were detected in both intestines (**a**, **c**), while in psA30P mice the ENS was found to be markedly stressed by the presence of lipid aldehydes in both, the SI (**b**) and the LI (**d**)

In order to determine the oxidative stress levels in psA30P mice we immunostained whole-mount muscle layers of psA0P mice and corresponding WT with malondialdehyde (MDA), a marker for lipid peroxidation representing oxidative stress, along with 4′,6-Diamidin-2-phenylindol (DAPI), and PGP 9.5. As illustrated in Fig. 7, there was no oxidative stress in terms of lipid peroxidation present in the WT (Fig. 7a,c), neither in the SI nor in the LI. In contrary, an increased expression of lipid aldehydes was located in the ENS ganglia in both, the SI (Fig. 7b) and the LI (Fig. 7d), respectively, giving evidence for oxidative stress in the gut of psA30P mice.

### Dysregulated miRNA expression in the ENS of psA30P mice

**Fig. 8:**
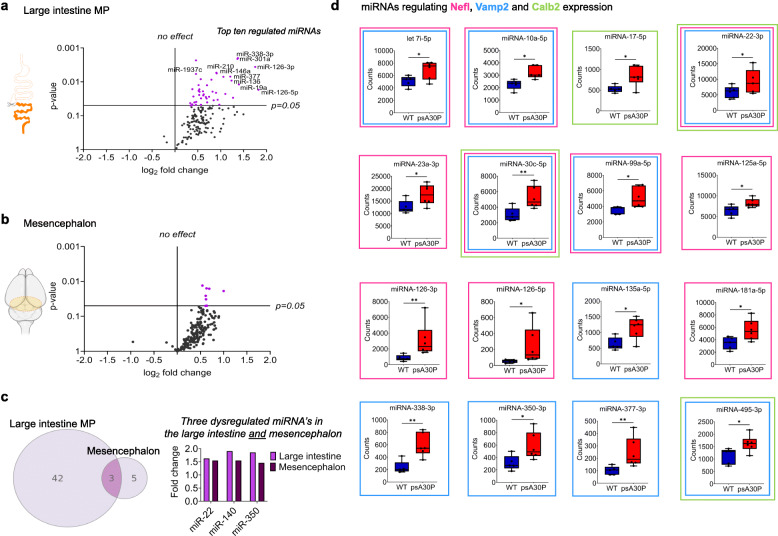
miRNA expression analysis of 2-month-old pre-symptomatic (ps)A30P mice. Volcano plots show miRNA profiling in the myenteric plexus (MP) of the large intestine (LI, **a**) and the mesencephalon (**b**) of psA30P mice compared with controls. Expression data for 578 miRNAs were generated by nCounter NanoString analysis. In total, 166 miRNAs were expressed in the MP of the LI (**a**) and 210 miRNAs in the mesencephalon (**b**) with a minimum of 100 counts. The left side represents the significance level of downregulated miRNAs and the right side the significance level of upregulated miRNAs. Purple circles indicate significantly dysregulated miRNAs; black circles indicate miRNAs whose expression was not significantly altered. The horizontal line in miRNA expression marks the threshold for the t-test *p* value (= 0.05). (**c**) In total, we identified 45 significantly upregulated miRNAs in the MP of the LI and eight upregulated miRNAs in the mesencephalon of psA30P mice. Among them, three are expressed in the MP of the LI and the mesencephalon, but with a higher fold change in the MP. (**d**) Box plots show regulated miRNAs in psA30P mice compared with wild types (WT) targeting neurofilament light chain (Nefl, pink framed boxes), vesicle-associated membrane protein 2 (Vamp2, blue framed boxes), and calbindin 2 (Calb2, green framed boxes). WT mice are shown in blue (n = 5), A30P mice in red (n = 6). Quantitative data are expressed as means ± SD using GraphPad Prism 8. * *p* ≤ 0.05 and ** *p* ≤ 0.01 using Student’s t test and Cohen’s d (Supplementary Table 7f)

To provide further knowledge into gene expression and regulation of pathologic pathways in the ENS during PD, we evaluated the possible regulating role of miRNAs. Therefore, total RNA was isolated from the MP of the LI and the mesencephalon, and was used to profile the miRNA spectrum in psA30P and WT mice by means of a NanoString nCounter® mouse expression assay (Supplementary Table 3). Individual Volcano plots were applied to identify differentially expressed miRNAs with statistically significance. As presented in the volcano plots, we found 166 robustly expressed miRNAs (more than 100 counts) in the MP of the LI in psA30P mice (Fig. 8a) and 210 miRNAs in the mesencephalon (Fig. 8b) compared with WT mice (Supplementary Tables 4 and 5). This dysregulation was statistically significant for 45 miRNAs in the MP (Fig. 8a), and for only eight miRNAs in the mesencephalon (Fig. 8b). Three miRNAs (miRNA-22, miRNA-140 and miRNA-350) were differentially expressed in both tissues, while the fold changes of these miRNA expressions were distinctly higher in the MP of the LI than in the mesencephalon (Fig. 8c).

The top ten regulated miRNAs in the MP of the LI with the highest fold change and the lowest *p*-value were following miRNAs: miRNA-19a, 126-3p, 126-5p, 136-5p, 146a-5p, 210-3p, 301a-3p, 338-3p, 377-3p, and 1937c (Supplementary Fig. 7 and Fig. 8a). In the MP of the LI we observed various significant expression changes for miRNAs that target Nefl, Vamp2, and Calb2, supporting our idea that these proteins are potential biomarkers for early-stage PD. Detailed examination of protein-miRNA clustering using mirWALK 3.0 [32] revealed that Nefl (Fig. 8d, pink framed boxes) and Vamp2 (Fig. 8d, blue framed boxes) are both targeted by ten miRNAs, and Calb2 (Fig. 8d, green framed boxes) is clustering with four miRNAs. All miRNAs were significantly upregulated, while the corresponding proteins were all significantly downregulated (Fig. 4f), thus implying a counter-rotated expression. Two miRNAs, miR-22-3p and miR-30c-5p, target all three selected proteins, Nefl, Vamp2 and Calb2.

### miRNA dysregulation is consistent in mice and men

**Fig. 9:**
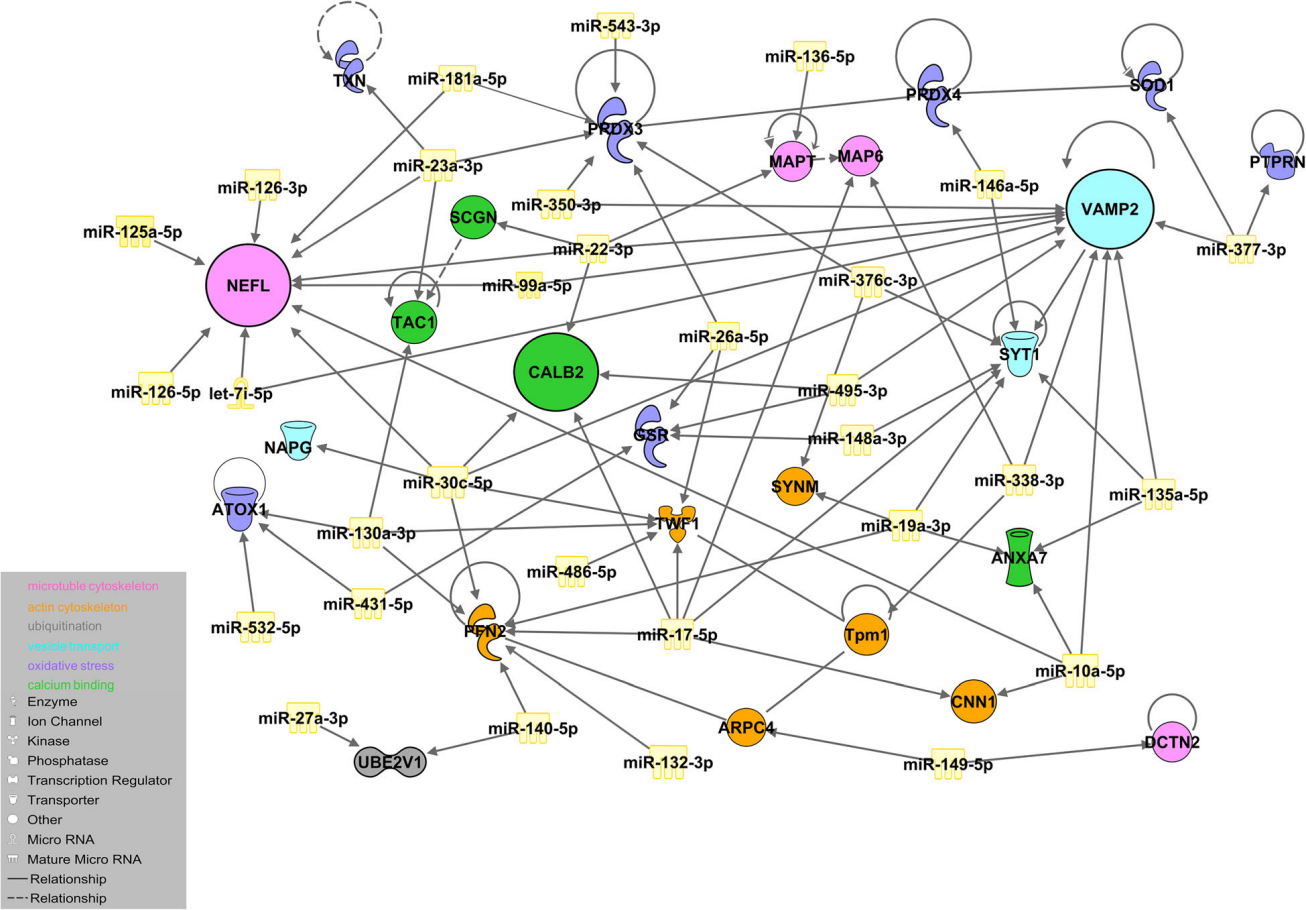
Ingenuity pathway analysis (IPA). IPA shows the relationships between altered miRNAs in the myenteric plexus (MP) of the large intestine (LI) from pre-symptomatic (ps)A30P mice compared with wild type (WT) controls and their target proteins. IPA functional pathway analysis was used to predict the top transcriptional regulators from differentially expressed proteins in murine PD models. miRNA target (protein) filter pairing analysis was done in order to identify co-regulated miRNAs/target pairs. We identified 77 protein-miRNA interactions which involved 25 dysregulated proteins and 31 miRNAs and eight protein-protein interactions. These predictions are based on Ingenuity Pathway Knowledge Base (IPKB). Nefl is targeted by let 7i-5p, miR-10a-5p, miR-22-3p, miR-23a-3p, miR-30c-5p, miR-99a-5p, miR-125a-5p, miR-126-3p, miR-126-5p, and miR-181a-5p; Vamp2 is targeted by let-7i-5p, miR-10a-5p, miR-22-3p, miR-30c-5p, miR-99a-5p, miR-135a-5p, miR-338-3p, miR-350-3p, miR-377-3p, and miR-495-3p; Calb2 is targeted by miR-17-5p, miR-22-3p, miR-30c-5p, and miR-495-3p

To determine the functional impact of miRNA dysregulation on PD-associated protein networks, we performed an IPA-based network analysis using all 45 upregulated miRNAs and all 49 altered proteins from our defined functional groups (Fig. 9). The majority of dysregulated miRNAs were involved in the same pathways as the proteins identified in our proteome analyses, such as protein ubiquitination, calcium signaling, cell and oxidative stress, synaptic transmission, and cytoskeleton assembly. These data revealed 77 protein-miRNA interactions, involving 25 proteins and 31 miRNAs, in which the proteins were downregulated and the respective miRNAs were upregulated, as well as eight protein-protein interactions. Nefl, Calb2, and Vamp2 clustered extensively with several miRNAs. Thirty-one miRNAs from all dysregulated miRNAs in psA30P mice have been shown to be dysregulated in human PD tissues and body fluids (Supplementary Table 6a), and 13 have been implicated in PD in mouse, rat, and in vitro models (Supplementary Table 6b). However, these miRNAs were only investigated during clinical stages of PD and in the CNS, and not during pre-symptomatic stages and in the gut or MP like in the present study. In addition, we detected 10 significantly dysregulated miRNAs in the MP of the LI in psA30P mice, which, to the best of our knowledge, have not been associated with PD to date (Supplementary Table 6c).

## Discussion

PD is currently diagnosed based on motor impairments that are only present during progressive stages of the disease [69]. These clinical symptoms are caused by Lewy bodies aggregating in the brain and by degeneration of dopaminergic neurons in the substantia nigra. Up to 60–70% of these neurons are already lost when the first motor symptoms appear [70]. The gold-standard treatment for managing these motor symptoms in advanced PD is oral administration of the dopamine precursor levodopa (L-dopa). Although short-term oral use of L-dopa is highly effective, long-term use is associated with several complications [71]. Thus, early diagnosis of PD combined with an efficient prophylaxis or therapy that delays or even prevents PD is essential. However, early diagnosis is challenging because biopsies cannot be easily obtained from the brain to monitor the progression of the disease over time. Early pathological signs of PD have been reported in the ENS of patients, suggesting that neurodegeneration may start in the gut and spreads to the brain [7, 8].

Recent studies gave evidence that PD might be separated into a PNS-first and a CNS-first subtype [72, 73]. The brain-first type is associated with a primary occurrence of the α-synuclein pathology in the brain and a secondary spread to the ENS. The body-first type is hallmarked by an initial contribution of the ENS, mostly accompanied by non-motoric symptoms, followed by a striatal dopaminergic dysfunction.

Based on this hypothesis, pathological analysis of the GI tract may detect PNS-first PD early enough to apply preventative therapies. In addition to motor impairments, bowel disturbances have been described in PD patients before clinical symptoms develop [74].

In our study we provide an assessment of ENS dysfunction early in PD pathogenesis in α-synuclein-overexpressing transgenic A30P mice, presumably mirroring the body-first PD type. To identify early biomarkers for PD within the gut, analyses were made using A30P mouse PD model, which allows an investigation of pre-symptomatic stages unlike in human patients. First we defined a pre-symptomatic (ps) stage of disease in which the mice neither exhibited any motoric impairments nor showed an imbalance of striatal dopamine levels, as it is reported to take place during the clinical peak of disease [25, 75]. When exploring the gait, psA30P mice were inconspicuous, and aside from that, they did not show reduced striatal dopamine levels [76], therefore signifying a not yet diseased brain. However, we demonstrated that these young mice have a diseased gut possessing several α-synuclein aggregates or Lewy bodies, in fact in both intestines. We propose that the ENS of α-synuclein-overexpressing transgenic A30P mice is more sensitive to A30P α-synuclein than the CNS, confirming the assumption, that the A30P mice either belong to the PNS-first PD category or that we do not yet understand that additional triggers might be responsible for the development of one or the other form of PD. The differences between the small and the large intestine may be caused by the presence of microbiota in the large intestine [14, 17]. This primary interface of microbial products with the ENS may trigger a prior start in the gut and also accounts for the advanced pathology in the large intestine compared with the small intestine. Hence, 2-month-old mice were specified as pre-symptomatic featuring a typical PD pathology in the gut, but not in the brain.

A major finding of the present study is decelerated GI motility in early PD, before motoric disturbances are detected. Consistent with these findings, PD patients with chronic constipation typically exhibit infrequent bowel movements, impaired propulsive colonic motility, and prolonged colonic transit with reduced rectal contractions [74]. Impaired colonic transit has already been described in young α-synuclein-overexpressing animals [77, 78], but besides the digestion speed no additional dynamic parameters were reported. In our ex vivo approach it is also possible to ascertain more convincing parameters, like number or velocity of individual contractions or dilatations respectively, as well as intervals. Additionally, it is known from biopsies that the LI is more affected by Lewy bodies in the myenteric neurons than the SI is [79, 80], reflecting our synuclein aggregate stainings. These observations support our finding of a stronger pathological effect in the LI than in the SI.

To provide evidence that the gut and ENS are involved in PD onset, and to confirm that PD pathogenesis starts in the gut, we investigated whether these functional changes correspond to molecular or morphological abnormalities in the GI tract at this stage. Changes in protein and miRNA expression in body fluid samples and post mortem brain biopsies from PD patients have improved our understanding of PD pathology. PD-related proteins are involved in several pathways including protein ubiquitination, oxidative stress, cytoskeleton development, synaptic function and vesicle release, and calcium binding [4]. Lowe et al. discovered that the majority of Lewy bodies in the brain contains ubiquitin [50], which conjugates to other proteins to bring about protein degradation [81]. In line with this, post mortem studies have shown defective proteasome activity in PD brains [82] that involve alterations in the ubiquitin-proteasome pathway [40]. To the best of our knowledge, dysregulation of proteins that regulate ubiquitination has not been investigated in the ENS during early stages of PD. In this study, we found that the expression of proteins, responsible for protein folding and ubiquitination, was altered in the MP of the SI and the LI. Of note, these proteins were all involved in regulating the ubiquitin-proteasome pathway [83].

Oxidative stress reflects the imbalance between the production and detoxification of reactive species, involving oxygen species and nitrogen species. At the cellular level, thiol redox homeostasis is maintained by the thioredoxin/peroxiredoxin (Trx/Prdx) pathway or by glutathione peroxidase (Gpx), which is part of the glutathione (Gsh) pathway. A marker for oxidative stress in PD postmortem brains is an increased level of hydroxyl radicals, which reduces expression of neuroprotective proteins like Trx1, glutathione-S-reductase (Gsr), and Gpx [84]. Moreover, reduced expression of Prdx in the brain has been linked to neurodegenerative diseases including Alzheimer’s disease and PD [85]. We found evidences for oxidative stress in the gut during early stages of PD in the present study. Expression of neuroprotective proteins was significantly altered in the MP of psA30P mice, for instance Gpx, Prdx2, Prdx4 and Gsr. These changes coincided with significantly increased expression of several miRNAs, which are known to regulate these proteins. It is well known that increased reactive oxygen species formation leads to tissue damage and its dysfunction induced oxidizing lipids which generate highly reactive lipid peroxidation products, such as peroxides and aldehydes. Massive amounts of malondialdehyde (MDA) can be found in the substantia nigra in the postmortem brain of PD patients [86]. In several independent studies elevated levels of MDA were demonstrated in the sera and plasma of PD patients [87–91]. Here, we also illustrated increased MDA levels outside the brain, e.g. the MP of young psA30P mice displaying enhanced oxidative stress in an early non-motoric stage of disease in the gut.

These findings provide the first proof that oxidative stress markers are strikingly dysregulated in the gut during early PD, and that these proteins may represent markers of early PD.

Cytoskeletal reorganization is required for neuronal homeostasis and plasticity, and for responses to axon injury and degeneration [35]. Disrupted post-translational modifications of neurofilament proteins have been linked to neurodegenerative disorders, including PD [56, 92].

Downregulation of neurofilament mRNA and proteins is common in the brain of PD patients and PD animal models [57, 58]. In agreement with these observations, we found significantly reduced Nefl protein expression in the ENS. In addition, we observed significant upregulation of miRNAs that regulate Nefl [32, 93]. Globular and filamentous actins are highly enriched in dendritic spines and are regulated during synaptic plasticity by actin-binding proteins (ABPs) [94]. ABPs, such as profilin and the actin-related-protein 2/3 complex (Arpc4), control actin polymerization and are dysregulated in neurological brain disorders including PD [95, 96]. Here, we showed that expression of profilin and Arpc4 is markedly changed in the LI of psA30P mice, indicating neurofilament disruption in the gut during early stages of PD. Interestingly, profilin is known to cluster with several miRNAs, e.g. miR-130a-3p and 132-3p, which were strongly upregulated in our psA30P model. Consistent with these observations, profilin expression was reduced in the MP of psA30P mice. In line with our findings, miR-132-3p expression was increased in the striatum of a symptomatic rotenone-induced PD rat model [96]. We present evidence that cytoskeletal disruption also contributes to neurodegeneration in the ENS during early stages of PD.

Dynactin is, as a part of the dynactin-dynein-complex (DDC), responsible for axonal transport, and the passage of cytoplasmic vesicles [97], and it affects α-synuclein aggregation increases Lewy body formation in different brain regions [98]. In this study, we found reduced dynactin 2 expression in the MP of psA30P mice, suggesting a reduced axonal transport of motor proteins in the gut at early stages of PD. This assumption is strengthened by upregulations of the counter-rotating miRNAs, which cluster to the DDC and regulate the same pathways [32, 99].

Different tau isoforms, like Mapt, are expressed in the CNS and ENS, but their expression is not altered in the ENS of PD patients with clinical symptoms [100]. Hallmark characteristics of prion diseases like PD are reduced synapse numbers and reduced dendritic spine densities. Boese et al. described a strong enrichment of several miRNAs including miR-136-5p, which targets Mapt, during the pre-clinical phase of prion disease [101]. Relevant to this, we report for the first time that Mapt is highly downregulated in the MP of the LI during the pre-symptomatic stage of PD. This downregulation correlated with an increased expression of miR-22-3p and miR-136-5p. Very recently, deletion of Map6, which interacts with Mapt [102] and was markedly decreased in our model, was reported to cause muscle weakness and atrophy, reduced calcium release, and alterations in the microtubule network, mirroring the early motoric impairments observed in the gut of α-synuclein-overexpressing mice [103, 104]. Interestingly, α-synuclein interacts with microtubules in HeLa cells [105] and preferably with tyrosinated tubulin in human mesencephalic neurons [106]. In agreement with our in vitro results, A30P α-synucleins regulate the microtubule network in PD. These mutated α-synucleins can affect neuronal microtubule assembly, and their overexpression leads to microtubule dysfunction and nigral neurite degeneration [107]. Our data imply that A30P α-synuclein pathology also impacts the cytoskeleton of ENS neurons during early PD.

Synaptic transmission is driven by Ca^2+^-dependent vesicle soluble N-ethylmaleimide-sensitive factor attachment (SNAREs), which allow synaptic vesicles to fuse to the plasma membrane [108]. Ca^2+^-binding synaptotagmins (Syt) interact with the SNARE proteins Vamp2, synataxin1 (Stx1), and synaptosomal-associated protein 25 (Snap25) for exocytosis [109]. Native α-synuclein, which is a neuronal pre-synaptic protein, is involved in the trafficking of synaptic vesicles and in vesicle exocytosis [110, 111]. However, aggregated α-synuclein is cytotoxic and mediates synaptic dysfunction by preventing the transport of synaptic proteins or the re-clustering of synaptic vesicles, resulting in a reduced neurotransmitter release [66, 112]. Misfolded α-synuclein co-localizes with Vamp2 and promotes SNARE-complex assembly [67, 113], while α-synuclein triple-knockout mice show neurological damage and reduced SNARE assembly [114]. In our study, Stx1, Syt1, and Vamp2, which are all involved in the synaptic vesicle cycle [115], were significantly downregulated in the gut of psA30P mice. This may explain the gut pathology observed in our psA30P mice supported by our findings that miRNAs clustering with Vamp2 are upregulated. This theory is also supported by the results of our in vitro experiments. Thus, the reduced expression of synaptic vesicle proteins, together with increased expression of the miRNAs that regulate them, may contribute to neuronal cell death in pre-symptomatic PD.

In addition to its important role in synaptic transmission, calcium also plays a pivotal role in neurodegeneration [116]. Intracellular Ca^2+^-dependent pathways dramatically increase calcium levels, which can trigger apoptotic cascades. Calcium-binding proteins (CaBPs) maintain calcium homeostasis for neuronal survival [117, 118]. CaBPs like calbindin-D (CalbD) and Calb2 are widely expressed in the CNS [119, 120] and the ENS [121–123]. Different neuronal populations have variable vulnerability to degeneration. Lack of CalbD in dopaminergic neurons increases susceptibility to PD; this is further supported by the low percentage of CalbD-positive neurons in advanced stages of PD [124]. Calb2 is expressed close to the plasma membrane and calcium channels [125], so may be involved in calcium influx, neuronal excitability, and neurotransmitter release [126, 127]. Moreover, Calb2 has been associated with other neurological disorders including schizophrenia [128]. Here, Calb2 positive neurons were paradoxically increased after acute α-synuclein exposure suggesting a temporary higher resistance of these neurons compared to other neuronal subtypes. However, a chronic exposure reduced Calb2 neurons and implicated an increase of miR-17-5p that regulates Calb2. It has been postulated, that an increase of Calb2 expression indicates neuroprotective processes in early stages of PD [68]. Hence, the increase of Calb2 in myenteric cultures might be a first reaction to A30P α-synuclein, while a reduction of Calb2 expression, as seen in whole-mount stainings of psA30P mice, resembles later stages of PD and chronic exposure to pathological A30P α-synuclein, where augmented cell death has already occurred. Furthermore, it has been reported, that an upregulation of miR-17-5p inhibits cell proliferation and induces apoptosis, while an inhibition induces neurite outgrowth [129]. Based on these findings, we postulate that Calb2 plays a crucial role in PD development in the gut. Our hypothesis is supported by previous findings that Calb2 expression is reduced in neurons following 6-OHDA treatment in a PD rat model [66, 67].

## Conclusions

In the present study we identified for the first time early functional and whole panel of molecular, gut-related biomarkers that may detect PD during early stages of the disease, long before motoric onset of the disease. Nefl, Vamp2, and Calb2, and their regulating miRNAs seem to be key players in the initiation of the disease. These proteins and miRNAs are mainly involved in cytoskeleton assembly, oxidative stress, ubiquitin-proteasome degradation, synaptic transmission, and calcium signaling, and may contribute to intestinal dysfunction during early PD. In postmortem brains and liquid biopsies from PD patients, which are derived from a very late clinical stage of the disease, markers like PINK1, Parkin, DJ1, and LRRK2 are dysregulated [130–132]. Hence, these are rather more suitable markers for later stages of the disease in the CNS. In the future, the ENS may serve as a source for minimally invasive gut biopsies for early detection of PD, which can be taken easily from human patients. Therefore, our novel biomarkers may open up the possibility of screening gut tissue to diagnose PD during its early stages, which may facilitate the timely treatment or even prevention of PD.

## Supplementary Information

**Additional file 1: Supplementary Figure 1.** Body mass of 2-month-old and 12–13-month-old A30P and wild type (WT) mice. **Supplementary Figure 2.** CatWalk XT analysis of 2-month-old and 12–13-month-old A30P and wild type (WT) mice. **Supplementary Figure 3.** Gut length of 2-month-old and 12–13-month-old A30P and WT mice. **Supplementary Figure 4.** STRING network analysis of 1044 proteins in the myenteric plexus (MP) of the small intestine (SI) and large intestine (LI). **Supplementary Figure 5**. Ganglionic areas in pre-symptomatic (ps)A30P and wild type (WT) mice. **Supplementary Figure 6.** Acute treatment of enteric cells with aggregated A30P α-synuclein. **Supplementary Figure 7.** Top ten regulated miRNAs in the myenteric plexus (MP) of the large intestine (LI) in pre-symptomatic (ps)A30P mice and wild type (WT) mice. **Supplementary Table 1.** Primary and secondary antibodies used and respective dilutions and applications. **Supplementary Table 2.** Protein expression profile in the myenteric plexus (MP) of the small intestine (SI) and the large intestine (LI) in pre-symptomatic (ps)A30P and wild type (WT) mice detected by mass spectroscopy. **Supplementary Table 3.** nCounter Mouse v1.5 miRNA Gene List. **Supplementary Table 4.** Expressed miRNAs in the mesencephalon of pre-symptomatic (ps)A30P and wild type (WT) mice. **Supplementary Table 5.** Expressed miRNAs in the myenteric plexus (MP) of the large intestine (LI) in pre-symptomatic (ps)A30P and wild type (WT) mice. **Supplementary Table 6**. Dysregulated miRNAs in different tissues and their correlation to Parkinson’s disease (PD) models and patients. **Supplementary Table 7.** List of Cohen’s d effect size [34] calculations.

**Additional file 2: Supplementary video 1.** Motility recordings during luminal perfusion of the small intestine in 2-month-old wild type mice.

**Additional file 3: Supplementary video 2.** Motility recordings during luminal perfusion of the small intestine in 2-month-old pre-sysmptomatic A30P mice.

**Additional file 4: Supplementary video 3.** Motility recordings during luminal perfusion of the large intestine in 2-month-old wild type mice.

**Additional file 5: Supplementary video 4.** Motility recordings during luminal perfusion of the large intestine in 2-month-old pre-sysmptomatic A30P mice.

## Data Availability

Raw images and datasets that support the findings of this study are available from the corresponding author upon reasonable request.

## Abbreviations

aA30P α-synuclein: aggregated A30P α-synuclein
ABP: Actin-binding protein
mA30P α-synuclein: monomeric A30P α-synuclein
Arpc4: Actin-related-protein 2/3 complex
BOS: Base of support
CaBP: Calcium-binding proteins
Calb2: Calbindin 2
CalbD: Calbindin-D
CNS: Central nervous system
CO: Control
DDC: Dynactin-dynein-complex
ENS: Enteric nervous system
GI: Gastrointestinal
Gpx: Glutathione peroxidase
Gsh: Glutathione
Gsr: Glutathione-S-reductase
hu-synuclein: human synuclein
LF: Left forepaw
LH: Left hindpaw
LI: Large intestine
Map 6: Microtubule-associated *protein* 6
Mapt: Microtubule associated protein tau
MDA: malondialdehyde
miRNA: MicroRNA
MP: Myenteric plexus
Nefl: Neurofilament light chain
PD: Parkinson’s disease
PGP 9.5: Protein gene product 9.5
Prx: Peroxiredoxin
psA30P: Pre-symptomatic A30P-α-synuclein mouse
RF: Right forepaw
RH: Right hindpaw
SI: Small intestine
Snap25: Synaptosomal-associated protein 25
SNARE: Vesicle soluble N-ethylmaleimide-sensitive factor attachment
Stx1: Synataxin1
Syt: Synaptotagmin
Trx: Thioredoxin
Tuj1: class III beta tubulin
Vamp2: Vesicle-associated membrane protein 2
WT: Wild type

## References

[1] Postuma RB, Berg D, Stern M, Poewe W, Olanow CW, Oertel W, et al. MDS clinical diagnostic criteria for Parkinson’s disease. Mov Disord. 2015;30(12):1591–601. 10.1002/mds.26424 26474316

[2] Cersosimo MG, Raina GB, Pecci C, Pellene A, Calandra CR, Gutiérrez C, et al. Gastrointestinal manifestations in Parkinson’s disease: prevalence and occurrence before motor symptoms. J Neurol. 2013;260(5):1332–8. 10.1007/s00415-012-6801-2 23263478

[3] Spillantini MG, Schmidt ML, Lee VMY, Trojanowski JQ, Jakes R, Goedert M. α-synuclein in Lewy bodies. Nature. 1997;388(6645):839–40. 10.1038/42166 9278044

[4] Hernandez DG, Reed X, Singleton AB. Genetics in Parkinson disease: Mendelian versus non-Mendelian inheritance. J Neurochem. 2016;139 Suppl 1(Suppl 1):59-74. 10.1111/jnc.13593 PMC5155439 27090875

[5] Braak H, De Vos RAI, Bohl J, Del Tredici K. Gastric α-synuclein immunoreactive inclusions in Meissner’s and Auerbach’s plexuses in cases staged for Parkinson’s disease-related brain pathology. Neurosci Lett. 2006;396(1):67–72. 10.1016/j.neulet.2005.11.012 16330147

[6] Lebouvier T, Chaumette T, Damier P, Coron E, Touchefeu Y, Vrignaud S, et al. Pathological lesions in colonic biopsies during Parkinson’s disease. Gut. 2008;57(12):1741–3. 10.1136/gut.2008.162503 19022934

[7] Braak H, Rüb U, Gai WP, Del Tredici K. Idiopathic Parkinson’s disease: possible routes by which vulnerable neuronal types may be subject to neuroinvasion by an unknown pathogen. J Neural Transm (Vienna). 2003;110(5):517–36. 10.1007/s00702-002-0808-2 12721813

[8] Braak H, Del Tredici K, Rüb U, De Vos RAI, Jansen Steur ENH, Braak E. Staging of brain pathology related to sporadic Parkinson’s disease. Neurobiol Aging. 2003;24(2):197–211. 10.1016/s0197-4580(02)00065-9 12498954

[9] Holmqvist S, Chutna O, Bousset L, Aldrin-Kirk P, Li W, Björklund T, et al. Direct evidence of Parkinson pathology spread from the gastrointestinal tract to the brain in rats. Acta Neuropathol. 2014;128(6):805–20. 10.1007/s00401-014-1343-6 25296989

[10] Challis C, Hori A, Sampson TR, Yoo BB, Challis RC, Hamilton AM, et al. Gut-seeded α-synuclein fibrils promote gut dysfunction and brain pathology specifically in aged mice. Nat Neurosci. 2020;23(3):327–336. 10.1038/s41593-020-0589-7 PMC7065967 32066981

[11] Van Den Berge N, Ferreira N, Gram H, Mikkelsen TW, Alstrup AKO, Casadei N (2019). Evidence for bidirectional and trans-synaptic parasympathetic and sympathetic propagation of alpha-synuclein in rats. Acta Neuropathol.

[12] Kim S, Kwon S, Kam T, Panicker N, Karuppagounder S, Lee S (2020). HHS Public Access.

[13] Scheperjans F, Aho V, Pereira PAB, Koskinen K, Paulin L, Pekkonen E, et al. Gut microbiota are related to Parkinson’s disease and clinical phenotype. Mov Disord. 2015;30(3):350–8. 10.1002/mds.26069 25476529

[14] Unger MM, Spiegel J, Dillmann KU, Grundmann D, Philippeit H, Bürmann J (2016). Short chain fatty acids and gut microbiota differ between patients with Parkinson’s disease and age-matched controls. Park Relat Disord.

[15] Endres K, Schäfer KH. Influence of commensal microbiota on the enteric nervous system and its role in neurodegenerative diseases. J Innate Immun. 2018;10(3):172–180. 10.1159/000488629 PMC6757170 29742516

[16] Seguella L, Sarnelli G, Esposito G. Leaky gut, dysbiosis, and enteric glia activation: the trilogy behind the intestinal origin of Parkinson’s disease. Neural Regen Res. 2020;15(6):1037–8. 10.4103/1673-5374.270308 PMC7034261 31823880

[17] Schwiertz A, Spiegel J, Dillmann U, Grundmann D, Bürmann J, Faßbender K, et al. Fecal markers of intestinal inflammation and intestinal permeability are elevated in Parkinson’s disease. Park Relat Disord. 2018;50:104–7. 10.1016/j.parkreldis.2018.02.022 29454662

[18] Zibert JR, Løvendorf MB, Litman T, Olsen J, Kaczkowski B, Skov L. MicroRNAs and potential target interactions in psoriasis. J Dermatol Sci. 2010;58(3):177–85. 10.1016/j.jdermsci.2010.03.004 20417062

[19] Mitchell PS, Parkin RK, Kroh EM, Fritz BR, Wyman SK, Pogosova-Agadjanyan EL, et al. Circulating microRNAs as stable blood-based markers for cancer detection. Proc Natl Acad Sci U S A. 2008;105(30):10513–8. 10.1073/pnas.0804549105 PMC2492472 18663219

[20] Gwiggner M, Martinez-Nunez RT, Whiteoak SR, Bondanese VP, Claridge A, Collins JE, et al. MicroRNA-31 and MicroRNA-155 are overexpressed in ulcerative colitis and regulate IL-13 signaling by targeting interleukin 13 receptor α-1. Genes (Basel). 2018;9(2):85. 10.3390/genes9020085 PMC5852581 29438285

[21] Dias V, Junn E, Mouradian MM. The role of oxidative stress in parkinson’s disease. J Parkinsons Dis. 2013;3(4):461–91. 10.3233/JPD-130230 PMC4135313 24252804

[22] De Guire V, Robitaille R, Tétreault N, Guérin R, Ménard C, Bambace N, et al. Circulating miRNAs as sensitive and specific biomarkers for the diagnosis and monitoring of human diseases: promises and challenges. Clin Biochem. 2013 ;46(10-11):846–60. 10.1016/j.clinbiochem.2013.03.015 23562576

[23] Mushtaq G, H. Greig N, Anwar F, A. Zamzami M, Choudhry H, M. Shaik M, et al. miRNAs as Circulating Biomarkers for Alzheimer’s Disease and Parkinson’s Disease. Med Chem (Los Angeles). 2016;12(3):217–25. 10.2174/1573406411666151030112140 PMC6138249 26527155

[24] Chen L, Yang J, Lü J, Cao S, Zhao Q, Yu Z. Identification of aberrant circulating miRNAs in Parkinson’s disease plasma samples. Brain Behav. 2018;8(4):e00941. 10.1002/brb3.941 PMC5893342 29670823

[25] Kahle PJ, Neumann M, Ozmen L, Müller V, Jacobsen H, Schindzielorz A, et al. Subcellular localization of wild-type and Parkinson’s disease-associated mutant α-synuclein in human and transgenic mouse brain. J Neurosci. 2000;20(17):6365–73. 10.1523/JNEUROSCI.20-17-06365.2000 PMC6772969 10964942

[26] Schäfer KH, Saffrey MJ, Burnstock G, Mestres-Ventura P. A new method for the isolation of myenteric plexus from the newborn rat gastrointestinal tract. Brain Res Protoc. 1997;1(2):109–13. 10.1016/s1385-299x(96)00017-7 9385071

[27] Grundmann D, Klotz M, Rabe H, Glanemann M, Schäfer KH. Isolation of high-purity myenteric plexus from adult human and mouse gastrointestinal tract. Sci Rep. 2015;5:9226. 10.1038/srep09226 PMC4366762 25791532

[28] Schreiber D, Jost V, Bischof M, Seebach K, Lammers WJ e., Douglas R, et al. Motility patterns of ex vivo intestine segments depend on perfusion mode. World J Gastroenterol. 2014;20(48):18216–27. 10.3748/wjg.v20.i48.18216 PMC4277959 25561789

[29] Mühlhaus T, Weiss J, Hemme D, Sommer F, Schroda M (2011). Quantitative shotgun proteomics using a uniform 15N-labeled standard to monitor proteome dynamics in time course experiments reveals new insights into the heat stress response of Chlamydomonas reinhardtii. Mol Cell Proteomics.

[30] Bossolani GDP, Pintelon I, Detrez JD, Buckinx R, Thys S, Zanoni JN, et al. Comparative analysis reveals Ce3D as optimal clearing method for in toto imaging of the mouse intestine. Neurogastroenterol Motil. 2019;31(5):e13560. 10.1111/nmo.13560 30761698

[31] Follmer C, Romão L, Einsiedler CM, Porto TCR, Lara FA, Moncores M, et al. Dopamine affects the stability, hydration, and packing of protofibrils and fibrils of the wild type and variants of α-synuclein. Biochemistry. 2007;46(2):472–82. 10.1021/bi061871+ 17209557

[32] Sticht C, De La Torre C, Parveen A, Gretz N. Mirwalk: An online resource for prediction of microrna binding sites. Plos One. 2018;13(10):e0206239. 10.1371/journal.pone.0206239 PMC6193719 30335862

[33] Cohen J (1988). 2.2. The effect size index: d. Statistical Power Analysis for the Behavioral Sciences.

[34] Pistacchi M, Gioulis M, Sanson F, de Giovannini E, Filippi G, Rossetto F, et al. Gait analysis and clinical correlations in early Parkinson’s disease. Funct Neurol. 2017;32(1):28–34. 10.11138/FNeur/2017.32.1.028 PMC5505527 28380321

[35] Muñoz-Lasso DC, Romá-Mateo C, Pallardó FV, Gonzalez-Cabo P. Much more than a scaffold: cytoskeletal proteins in neurological disorders. Cells. 2020;9(2):358. 10.3390/cells9020358 PMC7072452 32033020

[36] Bott CJ, Winckler B. Intermediate filaments in developing neurons: beyond structure. Cytoskeleton. 2020;77(3-4):110-128. 10.1002/cm.21597 31970897

[37] Muddapu VR, Dharshini SAP, Chakravarthy VS, Gromiha MM. Neurodegenerative diseases – is metabolic deficiency the root cause? Front In Neurosci. 2020;14:213. 10.3389/fnins.2020.00213 PMC7137637 32296300

[38] Bader V, Winklhofer KF. PINK1 and Parkin: team players in stress-induced mitophagy. Biol Chem. 2020;401(6–7):891–9. 10.1515/hsz-2020-0135 32297878

[39] Oliveira LOD, da Silva PIC, Filho RPR, Progênio RCS, de Oliveira VDPS, Silva RC, et al. Prior exercise protects against oxidative stress and motor deficit in a rat model of Parkinson’s disease. Metab Brain Dis. 2020;35(1):175–81. 10.1007/s11011-019-00507-z 31782038

[40] Lim KL, Tan JMM. Role of the ubiquitin proteasome system in Parkinson’s disease. BMC Biochem. 2007;8 Suppl 1(Suppl 1):S13. 10.1186/1471-2091-8-S1-S13 PMC2106364 18047737

[41] Ji T, Zhang X, Xin Z, Xu B, Jin Z, Wu J, et al. Does perturbation in the mitochondrial protein folding pave the way for neurodegeneration diseases? Ageing Res Rev. 2020;57:100997. 10.1016/j.arr.2019.100997 31816444

[42] Devi L, Raghavendran V, Prabhu BM, Avadhani NG, Anandatheerthavarada HK. Mitochondrial import and accumulation of α-synuclein impair complex I in human dopaminergic neuronal cultures and Parkinson disease brain. J Biol Chem. 2008;283(14):9089–100. 10.1074/jbc.M710012200 PMC2431021 18245082

[43] Petryszyn S, Parent A, Parent M. The calretinin interneurons of the striatum: comparisons between rodents and primates under normal and pathological conditions. J Neural Transm. 2018;125(3):279–90. 10.1007/s00702-017-1687-x 28168621

[44] Haberman A, Williamson WR, Epstein D, Wang D, Rina S, Meinertzhagen IA, et al. The synaptic vesicle SNARE neuronal synaptobrevin promotes endolysosomal degradation and prevents neurodegeneration. J Cell Biol. 2012;196(2):261–76. 10.1083/jcb.201108088 PMC3265959 22270918

[45] Choi BK, Choi MG, Kim JY, Yang Y, Lai Y, Kweon DH, et al. Large α-synuclein oligomers inhibit neuronal SNARE-mediated vesicle docking. Proc Natl Acad Sci USA. 2013;110(10):4087–92. 10.1073/pnas.1218424110 PMC3593925 23431141

[46] Soukup S, Vanhauwaert R, Verstreken P. Parkinson’s disease: convergence on synaptic homeostasis. EMBO J. 2018;37(18):e98960. 10.15252/embj.201898960 PMC6138432 30065071

[47] Pellegrini C, Ippolito C, Segnani C, Dolfi A, Errede M, Virgintino D, et al. Pathological remodelling of colonic wall following dopaminergic nigrostriatal neurodegeneration. Neurobiol Dis. 2020;139:104821. 10.1016/j.nbd.2020.104821 32088380

[48] Raja SA, Abbas S, Shah STA, Tariq A, Bibi N, Yousuf A, et al. Increased expression levels of syntaxin 1a and synaptobrevin 2/vesicle-associated membrane protein-2 are associated with the progression of bladder cancer. Genet Mol Biol. 2019;42(1):40–7. 10.1590/1678-4685-GMB-2017-0339 PMC6428126 30672978

[49] Langston J, Ballard P, Tetrud J, Irwin I. Chronic Parkinsonism in humans due to a product of meperidine-analog synthesis. Science. 1983;219(4587):979–80. 10.1126/science.6823561 6823561

[50] Lowe J, Blanchard A, Morrell K, Lennox G, Reynolds L, Billett M, et al. Ubiquitin is a common factor in intermediate filament inclusion bodies of diverse type in man, including those of Parkinson’s disease, Pick’s disease, and Alzheimer’s disease, as well as Rosenthal fibres in cerebellar astrocytomas, cytoplasmic bodies in m. J Pathol. 1988;155(1):9–15. 10.1002/path.1711550105 2837558

[51] Mayer RJ, Lowe J, Lennox G, Doherty F, Landon M. Intermediate filaments and ubiquitin: a new thread in the understanding of chronic neurodegenerative diseases. Prog Clin Biol Res. 1989;317:809–18. 2557642

[52] Przedborski S, Jackson-Lewis V. Mechanisms of MPTP toxicity. Mov Disord. 1998;13 Suppl 1:35–8. 9613716

[53] Cappelletti G, Maggioni MG, Maci R. Influence of MPP+ on the state of tubulin polymerisation in NGF- differentiated PC12 cells. J Neurosci Res. 1999;56(1):28–35. 10.1002/(SICI)1097-4547(19990401)56:1<28::AID-JNR4>3.0.CO;2-2 10213472

[54] Federico A, Cardaioli E, Da Pozzo P, Formichi P, Gallus GN, Radi E. Mitochondria, oxidative stress and neurodegeneration. J Neurol Sci. 2012;322(1-2):254–62. 10.1016/j.jns.2012.05.030 22669122

[55] Rahman AA, Morrison BE. Contributions of VPS35 mutations to Parkinson’s disease. Neuroscience. 2019;401:1–10. 10.1016/j.neuroscience.2019.01.006 PMC6422337 30660673

[56] Conde MA, Alza NP, Iglesias González PA, Scodelaro Bilbao PG, Sánchez Campos S, Uranga RM, et al. Phospholipase D1 downregulation by α-synuclein: implications for neurodegeneration in Parkinson’s disease. Biochim Biophys Acta - Mol Cell Biol Lipids. 2018;1863(6):639–50. 10.1016/j.bbalip.2018.03.006 29571767

[57] Hill WD, Arai M, Cohen JA, Trojanowski JQ. Neurofilament mRNA is reduced in Parkinson’s disease substantia nigra pars compacta neurons. J Comp Neurol. 1993;329(3):328–36. 10.1002/cne.903290304 8459049

[58] Wellings TP, Brichta AM, Lim R. Altered neurofilament protein expression in the lateral vestibular nucleus in Parkinson’s disease. Exp Brain Res. 2017;235(12):3695–708. 10.1007/s00221-017-5092-3 28929183

[59] Zhang XM, Anwar S, Kim Y, Brown J, Comte I, Cai H, et al. The A30P α-synuclein mutation decreases subventricular zone proliferation. Hum Mol Genet. 2019;28(14):2283–94. 10.1093/hmg/ddz057 PMC6606853 31267130

[60] Li J, Sun Y, Chen J. Transcriptome sequencing in a 6-hydroxydopamine rat model of Parkinson’s disease. Genes Genet Syst. 2019;94(2):61–9. 10.1266/ggs.18-00036 30713215

[61] Ma Y, Zhan M, OuYang L, Li Y, Chen S, Wu J, et al. The effects of unilateral 6-OHDA lesion in medial forebrain bundle on the motor, cognitive dysfunctions and vulnerability of different striatal interneuron types in rats. Behav Brain Res. 2014;266:37–45. 10.1016/j.bbr.2014.02.039 24613235

[62] Mura A, Feldon J, Mintz M. The expression of the calcium binding protein calretinin in the rat striatum: effects of dopamine depletion and L-DOPA treatment. Exp Neurol. 2000;164(2):322–32. 10.1006/exnr.2000.7441 10915571

[63] Trojanowski JQ, Lee VMY. Aggregation of neurofilament and α-synuclein proteins in Lewy bodies: implications for the pathogenesis of Parkinson disease and Lewy body dementia. Arch Neurol. 1998;55(2):151–2 10.1001/archneur.55.2.151 9482355

[64] Chen QQ, Haikal C, Li W, Li MT, Wang ZY, Li JY. Age-dependent alpha-synuclein accumulation and aggregation in the colon of a transgenic mouse model of Parkinson’s disease. Transl Neurodegener. 2018;7:13. 10.1186/s40035-018-0118-8 PMC6026335 29988485

[65] Gaugler MN, Genc O, Bobela W, Mohanna S, Ardah MT, El-Agnaf OM, et al. Nigrostriatal overabundance of α-synuclein leads to decreased vesicle density and deficits in dopamine release that correlate with reduced motor activity. Acta Neuropathol. 2012;123(5):653–69. 10.1007/s00401-012-0963-y 22361813

[66] Nemani VM, Lu W, Berge V, Nakamura K, Onoa B, Lee MK, et al. Increased Expression of α-Synuclein Reduces Neurotransmitter Release by Inhibiting Synaptic Vesicle Reclustering after Endocytosis. Neuron. 2010;65(1):66–79. 10.1016/j.neuron.2009.12.023 PMC3119527 20152114

[67] Choi MG, Kim MJ, Kim DG, Yu R, Jang YN, Oh WJ. Sequestration of synaptic proteins by alphasynuclein aggregates leading to neurotoxicity is inhibited by small peptide. Plos One. 2018;13(4):e0195339. 10.1371/journal.pone.0195339 PMC5880409 29608598

[68] Mouatt-Prigent A, Agid Y, Hirsch EC (1994). Does the calcium binding protein calretinin protect dopaminergic neurons against degeneration in Parkinson’s disease?. Brain Res.

[69] Brognara L, Palumbo P, Grimm B, Palmerini L. Assessing gait in Parkinson’s disease using wearable motion sensors: a systematic review. Diseases. 2019;7(1):18. 10.3390/diseases7010018 PMC6473911 30764502

[70] Lang AE, Lozano AM. Parkinson’s disease. First of two parts. N Engl J Med. 1998;339(15):1044–53. 10.1056/NEJM199810083391506 9761807

[71] Mulroy E, Bhatia KP. The Gut Microbiome: A Therapeutically Targetable Site of Peripheral Levodopa Metabolism. Mov Disord Clin Pract. 2019;6(7):547–8. 10.1002/mdc3.12828 PMC6749799 31538088

[72] Borghammer P, Van Den Berge N (2019). Brain-first versus gut-first Parkinson’s disease: a hypothesis. J Parkinsons Dis.

[73] Horsager J, Andersen KB, Knudsen K, Skjærbæk C, Fedorova TD, Okkels N (2020). Brain-first versus body-first Parkinson’s disease: a multimodal imaging case-control study. Brain..

[74] Fasano A, Visanji NP, Liu LWC, Lang AE, Pfeiffer RF. Gastrointestinal dysfunction in Parkinson’s disease. Lancet Neurol. 2015;14(6):625–39. 10.1016/S1474-4422(15)00007-1 25987282

[75] Masato A, Plotegher N, Boassa D, Bubacco L (2019). Impaired dopamine metabolism in Parkinson’s disease pathogenesis. Mol Neurodegener.

[76] Szego ÉM, Gerhardt E, Kermer P, Schulz JB (2012). A30P α-synuclein impairs dopaminergic fiber regeneration and interacts with L-DOPA replacement in MPTP-treated mice. Neurobiol Dis.

[77] Wang L, Magen I, Yuan PQ, Subramaniam SR, Richter F, Chesselet MF (2012). Mice overexpressing wild-type human alpha-synuclein display alterations in colonic myenteric ganglia and defecation. Neurogastroenterol Motil.

[78] Kuo Y-M, Nwankwo EI, Nussbaum RL, Rogers J, Maccecchini ML. Translational inhibition of α-synuclein by Posiphen normalizes distal colon motility in transgenic Parkinson mice. Am J Neurodegener Dis. 2019;8(1):1–15. eCollection 2019. PMC6420700 30906671

[79] Pouclet H, Lebouvier T, Coron E, Neunlist M, Derkinderen P. Lewy pathology in gastric and duodenal biopsies in Parkinson’s Disease. Movement Disorders. 2012;27(6):708. 10.1002/mds.24993 22649005

[80] Wakabayashi K, Takahashi H, Ohama E, Ikuta F. Parkinson’s disease: an immunohistochemical study of Lewy body-containing neurons in the enteric nervous system. Acta Neuropathol. 1990;79(6):581–3. 10.1007/BF00294234 1972853

[81] Walden H, Muqit MMK. Ubiquitin and Parkinson’s disease through the looking glass of genetics. Biochem J. 2017;474(9):1439–51. 10.1042/BCJ20160498 PMC5390927 28408429

[82] Furukawa Y, Vigouroux S, Wong H, Guttman M, Rajput AH, Ang L, et al. Brain proteasomal function in sporadic Parkinson’s disease and related disorders. Ann Neurol. 2002;51(6):779–82. 10.1002/ana.10207 12112087

[83] Lehtonen Š, Sonninen TM, Wojciechowski S, Goldsteins G, Koistinaho J. Dysfunction of cellular proteostasis in Parkinson’s disease. Front Neurosci. 2019;13:457. 10.3389/fnins.2019.00457 PMC6524622 31133790

[84] Sian J, Dexter DT, Lees AJ, Daniel S, Agid Y, Javoy-Agid F, et al. Alterations in glutathione levels in Parkinson’s disease and other neurodegenerative disorders affecting basal ganglia. Ann Neurol. 1994;36(3):348–55. 10.1002/ana.410360305 8080242

[85] Jian W, Wei X, Chen L, Wang Z, Sun Y, Zhu S, et al. Inhibition of HDAC6 increases acetylation of peroxiredoxin1/2 and ameliorates 6-OHDA induced dopaminergic injury. Neurosci Lett. 2017;658:114–20. 10.1016/j.neulet.2017.08.029 28823893

[86] Dexter DT, Carter CJ, Wells FR, Javoy-Agid F, Agid Y, Lees A (1989). Basal lipid peroxidation in Substantia Nigra is increased in Parkinson’s disease. J Neurochem.

[87] Naduthota RM, Bharath RD, Jhunjhunwala K, Yadav R, Saini J, Christopher R (2017). Imaging biomarker correlates with oxidative stress in Parkinson’s disease. Neurol India.

[88] Kilinç A, Yalçin AS, Yalçin D, Taga Y, Emerk K (1988). Increased erythrocyte susceptibility to lipid peroxidation in human Parkinson’s disease. Neurosci Lett.

[89] Kalra J, Rajput AH, Mantha SV, Chaudhary AK, Prasad K (1992). Oxygen free radical producing activity of polymorphonuclear leukocytes in patients with Parkinson’s disease. Mol Cell Biochem.

[90] Mythri RB, Venkateshappa C, Harish G, Mahadevan A, Muthane UB, Yasha TC (2011). Evaluation of markers of oxidative stress, antioxidant function and astrocytic proliferation in the striatum and frontal cortex of Parkinson’s disease brains. Neurochem Res.

[91] Navarro A, Boveris A, Bández MJ, Sánchez-Pino MJ, Gómez C, Muntané G (2009). Human brain cortex: mitochondrial oxidative damage and adaptive response in Parkinson disease and in dementia with Lewy bodies. Free Radic Biol Med.

[92] Shukla V, Skuntz S, Pant HC. Deregulated Cdk5 activity is involved in inducing Alzheimer’s disease. Arch Med Res. 2012;43(8):655–62 10.1016/j.arcmed.2012.10.015 PMC3532552 23142263

[93] Krämer A, Green J, Pollard J, Tugendreich S. Causal analysis approaches in ingenuity pathway analysis. Bioinformatics. 2014;30(4):523–30. 10.1093/bioinformatics/btt703 PMC3928520 24336805

[94] Welch MD, Iwamatsu A, Mitchison TJ. Actin polymerization is induced by Arp2/3 protein complex at the surface of listeria monocytogenes. Nature. 1997;385(6613):265–9. 10.1038/385265a0 9000076

[95] Bamburg JR, Bernstein BW. Actin dynamics and cofilin-actin rods in alzheimer disease. Cytoskeleton. 2016;73(9):477–97. 10.1002/cm.21282 PMC5345344 26873625

[96] Horst CH, Schlemmer F, de Aguiar Montenegro N, Domingues ACM, Ferreira GG, da Silva Ribeiro CY, et al. Signature of Aberrantly Expressed microRNAs in the Striatum of Rotenone-Induced Parkinsonian Rats. Neurochem Res. 2018;43(11):2132–40. 10.1007/s11064-018-2638-0 30267378

[97] Urnavicius L, Zhang K, Diamant AG, Motz C, Schlager MA, Yu M, et al. The structure of the dynactin complex and its interaction with dynein. Science. 2015;347(6229):1441–6. 10.1126/science.aaa4080 PMC4413427 25814576

[98] Shen C, Honda H, Suzuki SO, Maeda N, Shijo M, Hamasaki H, et al. Dynactin is involved in Lewy body pathology. Neuropathology. 2018;38(6):583–90. 10.1111/neup.12512 30215870

[99] Dweep H, Sticht C, Pandey P, Gretz N. MiRWalk - database: prediction of possible miRNA binding sites by “ walking” the genes of three genomes. J Biomed Inform. 2011;44(5):839–47. 10.1016/j.jbi.2011.05.002 21605702

[100] Kovacs GG, Robinson JL, Xie SX, Lee EB, Grossman M, Wolk DA, et al. Evaluating the patterns of aging-related tau astrogliopathy unravels novel insights into brain aging and neurodegenerative diseases. J Neuropathol Exp Neurol. 2017;76(4):270–88. 10.1093/jnen/nlx007 PMC6251691 28340083

[101] Boese AS, Saba R, Campbell K, Majer A, Medina S, Burton L, et al. MicroRNA abundance is altered in synaptoneurosomes during prion disease. Mol Cell Neurosci. 2016;71:13–24. 10.1016/j.mcn.2015.12.001 26658803

[102] Liu W, Wang X. Prediction of functional microRNA targets by integrative modeling of microRNA binding and target expression data. Genome Biol. 2019;20(1):18. 10.1186/s13059-019-1629-z PMC6341724 30670076

[103] Kuo YM, Li Z, Jiao Y, Gaborit N, Pani AK, Orrison BM, et al. Extensive enteric nervous system abnormalities in mice transgenic for artificial chromosomes containing Parkinson disease-associated α-synuclein gene mutations precede central nervous system changes. Hum Mol Genet. 2010;19(9):1633–50. 10.1093/hmg/ddq038 PMC2850613 20106867

[104] Sébastien M, Giannesini B, Aubin P, Brocard J, Chivet M, Pietrangelo L, et al. Deletion of the microtubule-associated protein 6 (MAP6) results in skeletal muscle dysfunction. Skelet Muscle. 2018;8(1):30. 10.1186/s13395-018-0176-8 PMC6147105 30231928

[105] Zhou RM, Huang YX, Li XL, Chen C, Shi Q, Wang GR, et al. Molecular interaction of α-synuclein with tubulin influences on the polymerization of microtubule in vitro and structure of microtubule in cells. Mol Biol Rep. 2010;37(7):3183–92. 10.1007/s11033-009-9899-2 19826908

[106] Cartelli D, Aliverti A, Barbiroli A, Santambrogio C, Ragg EM, Casagrande FVM, et al. α-Synuclein is a Novel Microtubule Dynamase. Sci Rep. 2016;6:33289. 10.1038/srep33289 PMC5024109 27628239

[107] Calogero AM, Mazzetti S, Pezzoli G, Cappelletti G. Neuronal microtubules and proteins linked to Parkinson’s disease: a relevant interaction? Biol Chem. 2019;400(9):1099–112. 10.1515/hsz-2019-0142 31256059

[108] McNew JA, Parlatl F, Fukuda R, Johnston RJ, Paz K, Paumet F, et al. Compartmental specificity of cellular membrane fusion encoded in SNARE proteins. Nature. 2000;407(6801):153–9. 10.1038/35025000 11001046

[109] Littleton JT, Chapman ER, Kreber R, Garment MB, Carlson SD, Ganetzky B. Temperature-sensitive paralytic mutations demonstrate that synaptic exocytosis requires SNARE complex assembly and disassembly. Neuron. 1998;21(2):401–13. 10.1016/s0896-6273(00)80549-8 9728921

[110] Murphy DD, Rueter SM, Trojanowski JQ, Lee VMY. Synucleins are developmentally expressed, and α-synuclein regulates the size of the presynaptic vesicular pool in primary hippocampal neurons. J Neurosci. 2000;20(9):3214–20. 10.1523/JNEUROSCI.20-09-03214.2000 PMC6773130 10777786

[111] Vargas KJ, Makani S, Davis T, Westphal CH, Castillo PE, Chandra SS. Synucleins regulate the kinetics of synaptic vesicle endocytosis. J Neurosci. 2014;34(28):9364–76. 10.1523/JNEUROSCI.4787-13.2014 PMC4087213 25009269

[112] Scott DA, Tabarean I, Tang Y, Cartier A, Masliah E, Roy S. A pathologic cascade leading to synaptic dysfunction in α-synuclein-induced neurodegeneration. J Neurosci. 2010;30(24):8083–95. 10.1523/JNEUROSCI.1091-10.2010 PMC2901533 20554859

[113] Burré J, Sharma M, Tsetsenis T, Buchman V, Etherton MR, Südhof TC. α-Synuclein promotes SNARE-complex assembly in vivo and in vitro. Science. 2010;329(5999):1663–7. 10.1126/science.1195227 PMC3235365 20798282

[114] Diao J, Burré J, Vivona S, Cipriano DJ, Sharma M, Kyoung M, et al. Native α-synuclein induces clustering of synaptic-vesicle mimics via binding to phospholipids and synaptobrevin-2/VAMP2. Elife. 2013;2:e00592. 10.7554/eLife.00592 PMC3639508 23638301

[115] Szklarczyk D, Gable AL, Lyon D, Junge A, Wyder S, Huerta-Cepas J, et al. STRING v11: protein-protein association networks with increased coverage, supporting functional discovery in genome-wide experimental datasets. Nucleic Acids Res. 2019;47(D1):D607–D613. 10.1093/nar/gky1131 PMC6323986 30476243

[116] Fairless R, Williams SK, Diem R. Dysfunction of neuronal calcium signalling in neuroinflammation and neurodegeneration. Cell Tissue Res. 2014;357(2):455–62. 10.1007/s00441-013-1758-8 24326615

[117] Persechini A, Moncrief ND, Kretsinger RH. The EF-hand family of calcium-modulated proteins. Trends Neurosci. 1989;12(11):462–7. 10.1016/0166-2236(89)90097-0 2479149

[118] Kennedy MB. Regulation of neuronal function by calcium. Trends Neurosci. 1989;12(11):417–20. 10.1016/0166-2236(89)90089-1 2479140

[119] Antal M, Freund TF, Polgár E. Calcium-binding proteins, parvalbumin- and calbindin-D 28k-immunoreactive neurons in the rat spinal cord and dorsal root ganglia: a light and electron microscopic study. J Comp Neurol. 1990;295(3):467–84. 10.1002/cne.902950310 2351764

[120] Foo KS, Hellysaz A, Broberger C. Expression and colocalization patterns of calbindin-D28k, calretinin and parvalbumin in the rat hypothalamic arcuate nucleus. J Chem Neuroanat. 2014;61–62:20–32. 10.1016/j.jchemneu.2014.06.008 25014433

[121] Timmermans JP, Adriaensen D, Cornelissen W, Scheuermann DW. Structural organization and neuropeptide distribution in the mammalian enteric nervous system, with special attention to those components involved in mucosal reflexes. Comp Biochem Physiol - A Physiol. 1997;118(2):331–40. 10.1016/s0300-9629(96)00314-3 9366065

[122] Furness JB. Types of neurons in the enteric nervous system. J Auton Nerv Syst. 2000;81(1–3):87–96. 10.1016/s0165-1838(00)00127-2 10869706

[123] Quinson N, Robbins H, Clark M, Furness J. Calbindin immunoreactivity of enteric neurons in the guinea-pig ileum. Cell Tissue Res. 2001;305(1):3–9. 10.1007/s004410100395 11512670

[124] Hirsch EC. Why are nigral catecholaminergic neurons more vulnerable than other cells in Parkinson’s disease? Ann Neurol. 1992;32 Suppl:S88–93. 10.1002/ana.410320715 1510386

[125] Hack NJ, Wride MC, Charters KM, Kater SB, Parks TN. Developmental changes in the subcellular localization of calretinin. J Neurosci. 2000;20(7):RC67. 10.1523/JNEUROSCI.20-07-j0001.2000 PMC6772258 10729356

[126] Pangrsic T, Gabrielaitis M, Michanski S, Schwaller B, Wolf F, Strenzke N, et al. EF-hand protein Ca2+ buffers regulate Ca2+ influx and exocytosis in sensory hair cells. Proc Natl Acad Sci USA. 2015;112(9):E1028–37. 10.1073/pnas.1416424112 PMC4352837 25691754

[127] Christel CJ, Schaer R, Wang S, Henzi T, Kreiner L, Grabs D, et al. Calretinin regulates Ca2+−dependent inactivation and facilitation of Cav2.1 Ca2+ channels through a direct interaction with the $α$12.1 subunit. J Biol Chem. 2012;287(47):39766–75. 10.1074/jbc.M112.406363 PMC3501022 23033479

[128] Gao R, Penzes P. Common Mechanisms of Excitatory and Inhibitory Imbalance in Schizophrenia and Autism Spectrum Disorders. Curr Mol Med. 2015;15(2):146–67. 10.2174/1566524015666150303003028 PMC4721588 25732149

[129] Dai H, Wang C, Yu Z, He D, Yu K, Liu Y, et al. MiR-17 regulates prostate Cancer cell proliferation and apoptosis through inhibiting JAK-STAT3 signaling pathway. Cancer Biother Radiopharm. 2018;33(3):103–109. 10.1089/cbr.2017.2386 29641255

[130] Abbas N, Lücking CB, Ricard S, Dürr A, Bonifati V, De Michele G, et al. A wide variety of mutations in the parkin gene are responsible for autosomal recessive parkinsonism in Europe. Hum Mol Genet. 1999;8(4):567–74. 10.1093/hmg/8.4.567 10072423

[131] Valente EM, Abou-Sleiman PM, Caputo V, Muqit MMK, Harvey K, Gispert S, et al. Hereditary early-onset Parkinson’s disease caused by mutations in PINK1. Science. 2004;304(5674):1158–60. 10.1126/science.1096284 15087508

[132] Di Fonzo A, Rohé CF, Ferreira J, Chien HF, Vacca L, Stocchi F, et al. A frequent LRRK2 gene mutation associated with autosomal dominant Parkinson’s disease. Lancet. 2005;365(9457):412–5 10.1016/S0140-6736(05)17829-5 15680456

